# Bored at home?—A systematic review on the effect of environmental enrichment on the welfare of laboratory rats and mice

**DOI:** 10.3389/fvets.2022.899219

**Published:** 2022-08-18

**Authors:** Paul Mieske, Ute Hobbiesiefken, Carola Fischer-Tenhagen, Céline Heinl, Katharina Hohlbaum, Pia Kahnau, Jennifer Meier, Jenny Wilzopolski, Daniel Butzke, Juliane Rudeck, Lars Lewejohann, Kai Diederich

**Affiliations:** ^1^German Center for the Protection of Laboratory Animals (Bf3R), German Federal Institute for Risk Assessment (BfR), Berlin, Germany; ^2^Institute of Animal Welfare, Animal Behavior and Laboratory Animal Science, Freie Universität Berlin, Berlin, Germany

**Keywords:** animal behavior, animal welfare, enriched environment, boredom, abnormal behavior, impoverished environment, laboratory animals (mouse and rat)

## Abstract

Boredom is an emotional state that occurs when an individual has nothing to do, is not interested in the surrounding, and feels dreary and in a monotony. While this condition is usually defined for humans, it may very well describe the lives of many laboratory animals housed in small, barren cages. To make the cages less monotonous, environmental enrichment is often proposed. Although housing in a stimulating environment is still used predominantly as a luxury good and for treatment in preclinical research, enrichment is increasingly recognized to improve animal welfare. To gain insight into how stimulating environments influence the welfare of laboratory rodents, we conducted a systematic review of studies that analyzed the effect of enriched environment on behavioral parameters of animal well–being. Remarkably, a considerable number of these parameters can be associated with symptoms of boredom. Our findings show that a stimulating living environment is essential for the development of natural behavior and animal welfare of laboratory rats and mice alike, regardless of age and sex. Conversely, confinement and under-stimulation has potentially detrimental effects on the mental and physical health of laboratory rodents. We show that boredom in experimental animals is measurable and does not have to be accepted as inevitable.

## Introduction

Recommendations for the husbandry of laboratory animals have been developed primarily with a view to standardizing experimental conditions and providing basic needs like water and food (1, 2). While satisfying basic needs helps avoid obvious pain and suffering in laboratory animals, in modern animal husbandry, saving resources and personnel costs is certainly also an important factor. For the planning of animal experiments, compromises are made between the various interests of researchers, animal caretakers, animal house managers, and animal welfare advocates. The guidelines of the EU-directive for example contains basic recommendations including that social animals should be kept in groups and that all laboratory animals should be given the opportunity to develop a wide range of normal behavior by providing a housing condition with sufficient complexity (Directive 2010/63/EU). Moreover, species-specific recommendations for rats and mice call for the provision of environmental enrichment to make laboratory animal housing more diverse (e.g., https://www.nc3rs.org.uk/3rs-resources/housing-and-husbandry-mouse). However, the type of housing referred to as “enriched environment” has changed significantly in the last decades (3, 4). For example, some of what was described as enriched animal husbandry 25 years ago nowadays just meets the basic recommendations [i.e., a cardboard tube (5, 6)]. Moreover, not only has the concept of enrichment changed over time, but so has the related conventional housing, which usually reflects the actual state of housing and legal requirements at the time of publication. Still, the current housing of most laboratory animals reflects an impoverished environment compared to truly species-specific housing. More specifically, one must assume that the lack of stimuli has far-reaching consequences for the well-being and health status of laboratory animals. In fact, Cait et al. (7) showed in a meta-analysis of 214 studies that conventional housing increases morbidity and mortality in research rodents. This is backed up by the here reviewed research on comparing laboratory conventional housing to a more varied enriched housing using more space, social contact, and/or physical items, which conclusively describe positive effects on well–being and behavior of mice provided with enrichment.

Environmental enrichment was initially introduced to laboratory animals for studies investigating the effect of environment on neurobiological parameters and learning behavior (8). For this very purpose it is still being used, for example, enrichment has been proven to be an effective therapeutic intervention in animal models of various diseases including stroke (9) and neurodegenerative diseases like Alzheimer's disease (10). Moreover, a stimulating environment improves learning and memory formation and is a potent trigger for neuroplastic events in the adult brain—a process originally thought to occur only in the young developing brain (11). In addition to disease models and neurobiological studies, increasing focus has been placed on the effect of stimulating environments on animal welfare. Stress-responses were mitigated under enriched housing conditions and the activity of natural-killer cells was enhanced (12). Expression of abnormal repetitive behaviors (i.e., stereotypies) were reduced in mice living in an enrichment environment (13–16) as were behavioral measures related to anxiety (13, 17). In summary, most publications indicate that enriched and varied housing conditions improve the well–being of laboratory animals. However, due to the low stimuli of conventional housing systems compared to a species-appropriate environment, this conclusion might be validly expressed in the opposite sense, that confined housing of laboratory animals compromises animal welfare and health.

Conventional husbandry of laboratory animals in research laboratories is characterized by confinement, monotony, and lack of challenge. In humans, such conditions are usually accompanied by a condition known as boredom. Boredom is an emotional state that usually relates to individuals having nothing to do, are not interested in their surroundings, and feel that life is dull and tedious (18, 19). This state could also very aptly describe the life of many laboratory animals housed in small barren cages. Few studies have directly addressed the issue of animal boredom so far. However, based on the findings from human studies (20), some behavioral abnormalities observed in captive animals can be readily linked to boredom (21).

For example, barbering behavior in animals has recently been related to Trichotillomania (“hair-pulling disorder”), a human disorder reportedly triggered by boredom (22, 23). Common abnormalities in captive animals are stereotypies, which are often related to a lack of stimulation in laboratory animals. Stereotypic behavior in mice like wire gnawing/bar- mouthing (6), circling at the cage lid, back-flipping, route tracing, and twirling (13, 14) was shown to be decreased under more stimulating enriched housing conditions. Another symptom of human boredom is an altered perception of time, in which time does not seem to pass in monotonous situations (24). In animals, this phenomenon can be measured objectively by training them to expect a specific event or reward after a predictable period and measuring their anticipatory behavior after being exposed to monotonous tasks or environments (21). This method was successfully trained in starlings using pecking a key as an anticipatory behavior (25). It is reasonable to assume that laboratory rodents also experience such a perceptual shift, but as far as we know this has not been investigated until now. Overall, it is not unfounded to speculate that the great overlap between human symptoms of boredom and similar phenomena in rodents indeed indicates that boredom in animals is both real and underestimated in laboratory animals.

Since a sufficient form of stimulation is lacking in boring situations, sensation-seeking or stimulus-seeking behavior also occurs in animals (21). This is seen as a form of escape from the unpleasant, boring situation. Indeed, it has been described that it is sometimes of little importance whether the stimulus has a positive or negative valence if interaction is possible at all (26). Burn et al. (27) showed stimulus seeking in ferrets as increased contact to negative and ambiguous stimuli compared to a control group which were provided a 1 h daily play time. Furthermore, ferrets without playtime spent more time lying awake with their eyes open, screeched more but sat and stood less, than after playtime (27). This form of awake inactivity as a form of suboptimal arousal can be seen as an indicator of bored animals as well and was also more apparent under non–stimulating housing conditions in mink (26, 28) and mice (29). Moreover, Meagher et al. (28) found increased interest in different external stimuli in mink in non-enriched environments as a form of sensation seeking of potentially bored animals. These two almost opposite extremes of boredom symptomatology—sensation seeking vs. awake inactivity—illustrate the multifaceted nature of the expression of boredom and thus the difficult search for a fixed definition for this distressing and damaging emotional condition. In psychology and medicine, boredom is gaining increasing recognition as a potentially harmful emotional state and as a field of research for translational studies (19, 30). Regarding animal welfare, boredom becomes a serious concern with an urgent need for research. In this systematic review, we therefore examined the literature on enriched environment with specific regard to the effects of housing conditions on well-being in laboratory mice and rats. Moreover, we examine the existing body of literature specifically related to boredom symptoms. By identifying measures of boredom as well as clues to potential cures for boredom in laboratory rodents, we aim to lay the groundwork for addressing this pressing issue in the context of modern animal research.

## Materials and methods

### Search strategy

In accordance with PRISMA guidelines, we searched the database Web of Science on July 5^*th*^, 2019, and again on February 24^*th*^, 2021, before data analysis commenced. We performed a supplementary search on Web of Science, Embase, and PubMed on March 29^th^, 2022. In terms of population, we focused on mice and rats, the most widely used laboratory animals in experimental research. Enriched housing conditions were included as intervention and a corresponding non-enriched/conventional housing as a comparator. At least one behavioral observation or test should have been performed as an outcome parameter for animal welfare. For further specialization of the resulting search string boredom and its synonyms were as well–included as their respective counterpart. To achieve a high outcome of relevant research papers in the final search, truncations with wildcards and synonyms were used in the search string establishment.

Searchstring:

*TS* = (boredom OR tedium OR ennui OR tediousness OR stuffiness OR dullness OR boringness OR monotony OR bor^*^ OR monoton^*^ OR motivat^*^ OR stimulat^*^ OR excit^*^ OR activ^*^ OR “affective state^*^”*)*

*AND TS* = (hous^*^ OR husbandry OR “animal keeping” OR environment^*^)

*AND TS* = *(mice OR mouse OR rat OR rats)*

*AND TS* = (behavior^*^ OR behavior^*^)

*AND TS* = *(standard OR conventional OR barren OR restricted OR impoverished)*

*AND TS* = (enrich^*^ *OR seminatural OR semi-naturalistic)*

### Selection of studies and information extraction

Abstract screening was done by nine reviewers (PM, UH, CF-T, CH, KH, PK, JM, JW, and KD) using the systematic reviewing online tool SyRF (http://www.syrf.org.uk/). Exclusion criteria included the use of other animals than rats and mice, no behavioral observation or experiment, use of only one housing condition, use of psychoactive drugs, use of a disease or transgenic models. We excluded editorials, conference abstracts, and review papers.

Ten reviewers (PM, UH, CF-T, CH, KH, PK, JM, JW, LL, and KD) independently screened full text and extracted information from eligible studies into a standardized form. Extracted parameters included species, strain, sex, age at the start of the housing period and the beginning of the behavioral experiment, the presence of a focus on animal welfare, the disease/lesion model, genetic modification, psychoactive substances/stimulations, enrichment category (social, object, space of home cage) and description, number of groups including control group and their housing, the mean behavioral outcome parameter and the used behavioral test. Compliance with scientific quality criteria in the included studies was assessed by ascertaining whether the allocation of animals to experimental groups was randomized and the assessment of outcomes was blinded. Any discrepancies were resolved by consensus. Randomization was done with the sample() function in the statistical computing software R (https://www.r-project.org/).

### Categorization and classification of age and durations of housing

Outcome parameters were categorized as follows: social behavior, aggressive behavior, abnormal behavior, affective well–being, activity, cognition, nociception, motor function, circadian rhythm, and exploratory behavior. An overview of the behavioral tests used in the studies and the assignment to the categories is shown in Supplementary Table 1. In addition, we extracted information about glucocorticoid hormones to evaluate effects of housing on stress. However, determination of stress hormones regarding sample source, number, and sampling-time was very heterogeneous. We therefore included glucocorticoids only in the main overview.

For a detailed examination of the effects of enrichment on animal welfare, the results of each study were considered in terms of sex of experimental animals, age of experimental animals, and duration of housing in the respective housing environments. For age classification, animals were designated as postnatal from 0 to 21 days of age, adolescent from 21 to 60 days of age, adult from 60 to 750 days of age, and post reproductive from more than 750 days of age (31). Duration of husbandry was classified in short, mid, and long-term housing duration with short defined as 0 to 30 days, mid with 30–90 days and long-term with more than 90 days.

For an in-depth investigation of boredom, all selected publications were screened again for boredom-specific parameters. Because few studies have explicitly examined boredom in animals, especially laboratory animals, the classification of boredom parameters was based on the symptomatology of human boredom and relevant translatable phenomena in mice and rats. The sources for these parameters were literature on human boredom (20, 32) and Charlotte Burn's pioneering review article on animal boredom (21). All studies selected in this systematic review were examined regarding these parameters. For the examination of the parameter “drug seeking behavior”, the studies related to the use of psychoactive substances that were excluded for the main analysis were re-integrated into this single analysis. Results of this part of the analysis are summarized in Table 1.

**Table 1:** Overview of publications addressing boredom related parameters, the respective outcome, and the behavioral test used.

**Boredom related parameter**	**Publications**	**Outcome**	**Behavioral test**
novelty seeking behavior	(33) (34) (35) (36) (37) (38) (39) (40) (41) (42) (43) (44) (45) (46) (47) (48) (49) (50) (51) (52) (53) (54) (55) (56) (57) (58) (59) (60) (61) (62) (63) (64) (65)	increase increase increase increase increase decrease decrease decrease decrease decrease increase increase increase increase increase increase increase decrease increase increase increase increase decrease increase increase increase increase decrease increase decrease increase decrease decrease	open field, behavioral observation Y-maze open field, light-dark test open field, behavioral observation open field, object recognition test elevated plus maze, open field open field activity cage open field, behavioral observation two-lever operant conditioning chamber object recognition test, open field behavioral observation radial-arm maze open field, Y-maze open field, Y-maze, light-dark test Y-maze, object recognition test barrier test, group test, intruder test open field, light-dark test elevated plus maze, light-dark test, concentric square field test Y-maze, light-dark test object recognition test corridor field task behavioral observation, elevated plus maze open field open field elevated plus maze, light-dark test hole board test light-dark test, concentric square field test open field, elevated plus maze open field, light-dark test, hole board test novelty place preference object recognition, passive avoidance test elevated plus maze, open field lever-responding task
depressive like behavior	(66) (67) (68) (69) (70) (71) (72) (73) (74) (75) (76) (77) (78) (79) (50) (80) (81) (82) (83) (29) (84) (85) (86)	decrease increase neutral decrease decrease decrease neutral decrease neutral increase increase increase neutral decrease decrease decrease decrease decrease decrease decrease decrease decrease decrease	forced swim test forced swim test forced swim test forced swim test forced swim test forced swim test tail suspension test forced swim test forced swim test tail suspension test forced swim test, sucrose preference forced swim test forced swim test forced swim test forced swim test, sucrose preference tail suspension test forced swim test forced swim test forced swim test forced swim test forced swim test sucrose preference forced swim test
drug-seeking behavior	(87) (88) (89) (90) (91) (92) (93) (94) (95) (96) (97) (98) (84) (99) (100) (101) (102) (103) (104) (105) (106) (107) (108) (65)	decrease decrease decrease decrease decrease decrease decrease decrease decrease decrease decrease decrease decrease decrease decrease decrease neutral decrease decrease decrease decrease neutral decrease decrease	conditioned place preference conditioned place preference conditioned place preference conditioned place preference cocaine context renewal test operant conditioning chamber conditioned place preference conditioned place preference operant conditioning chamber conditioned place preference drinking in the dark test operant conditioning chamber two-bottle choice test operant conditioning chamber conditioned place preference operant conditioning chamber operant conditioning chamber context induced relapse test conditioned place preference operant conditioning chamber liquid consumption sign tracking conditioned place preference alcohol self-administration
stereotypic behavior	(6) (109) (110) (44) (111) (112) (113) (114) (76) (115) (116) (29) (117) (118) (13) (16)	decrease decrease decrease neutral decrease decrease neutral decrease decrease neutral decrease decrease decrease neutral decrease decrease	behavioral observation behavioral observation behavioral observation behavioral observation behavioral observation behavioral observation behavioral observation behavioral observation activitymeter, behavioral observation behavioral observation behavioral observation behavioral observation activity testing chamber behavioral observation behavioral observation behavioral observation
motivation for stimulation	(119) (120) (121) (50) (122)	increase increase decrease increase decrease	running wheel, open field operant training operant conditioning test open field, hole board behavioral observation
Inactive but awake	(110) (123) (29) (124)	decrease decrease decrease decrease	behavioral observation open field, behavioral observation behavioral observation behavioral observation, open field
risk proneness	(125) (51) (126)	increase increase neutral	open field, radial water maze elevated plus maze, light-dark test open field, elevated plus maze, inhibitory avoidance

The table is sorted showing the boredom related behaviors with the largest number of publications first. The publications investigating the specific behaviors are sorted by year ascending in order to show actual trends in this field of research. The boredom related parameters escape behavior, hair pulling and time perception were not investigated in the reviewed publications.

### Analysis

Analysis and illustrations were done using the software environment R (version 3.6.3, https://www.r-project.org/, R Foundation for Statistical Computing, Vienna, Austria) and the development software and graphical user interface RStudio (version 1.2.1,335, RStudio, Inc., Boston, MA, United States).

To assess the impact of enrichment on the defined categories, it was determined whether the selected studies reported an increase or a decrease in the respective categories; if no change was found, the result was classified as neutral. In the figures, the bars represent the studies that reported an increase, a decrease, or no change in the respective parameter in the corresponding category. The thickness of the bars reflects the amount of identified and investigated studies for this category. The numbers indicate the observed effect of the enrichment as a decimal number. If this value reaches 1, all studies in this category have observed an increase; correspondingly, a decrease if the value reaches−1. A bar located further to the right of the scale thus indicates an increasing effect of the applied enrichment on the category under consideration. The numbers correspond to the principle of a Likert scale.

## Results

### Study inclusion and study characteristics

**Figure 1:**
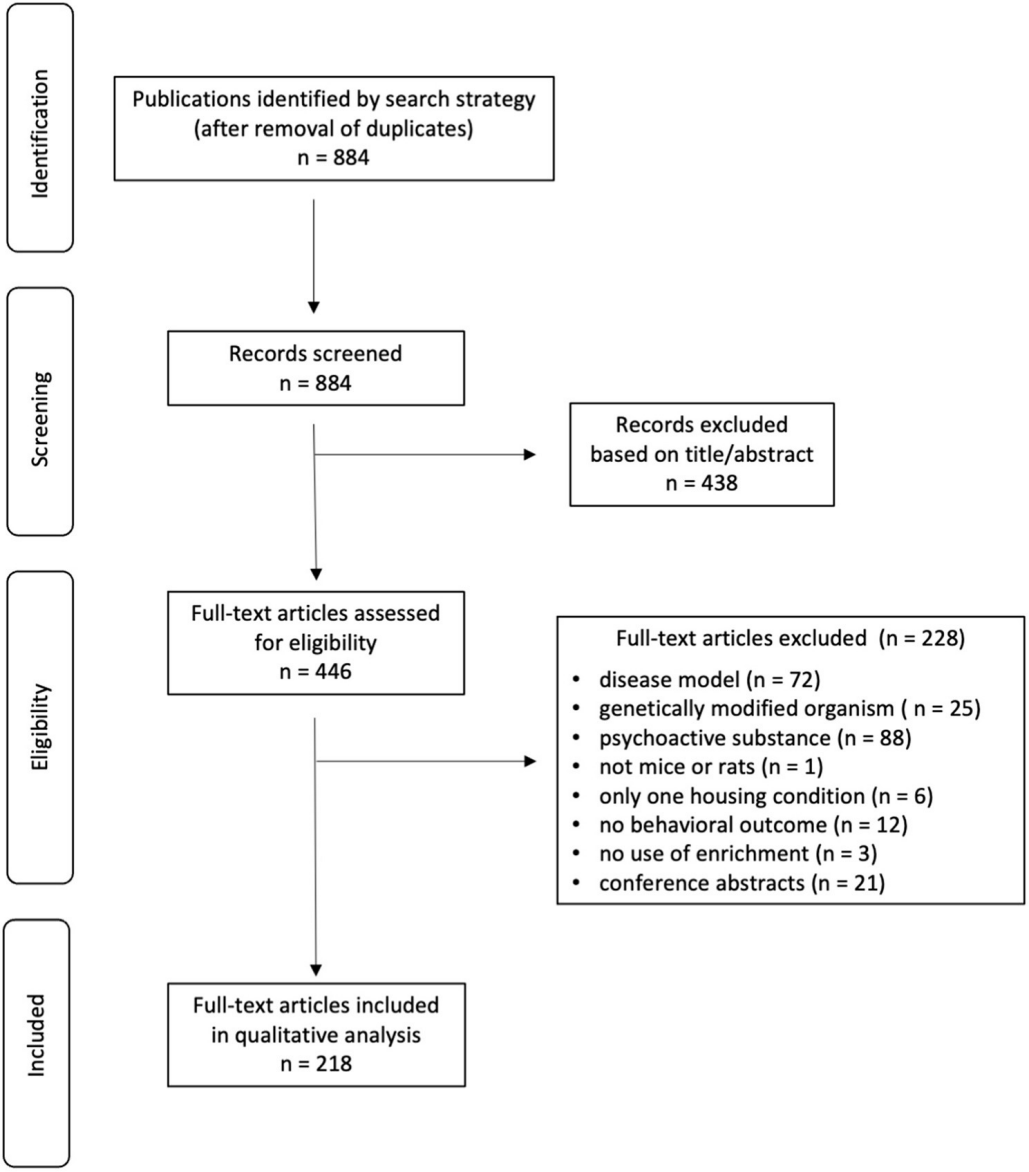
PRISMA flow diagram of article identification and selection.

Search strategy and study selection results are presented in Figure 1. After removal of duplicates, 884 titles/abstracts were screened, of which 438 were excluded. Full texts of the remaining 446 records were then screened, and 228 did not meet the eligibility criteria. This left 186 articles for qualitative synthesis.

71.6% of studies reported randomization of animals to treatment groups and only 24.3% of studies indicated blinding of outcome assessors.

**Figure 2:**
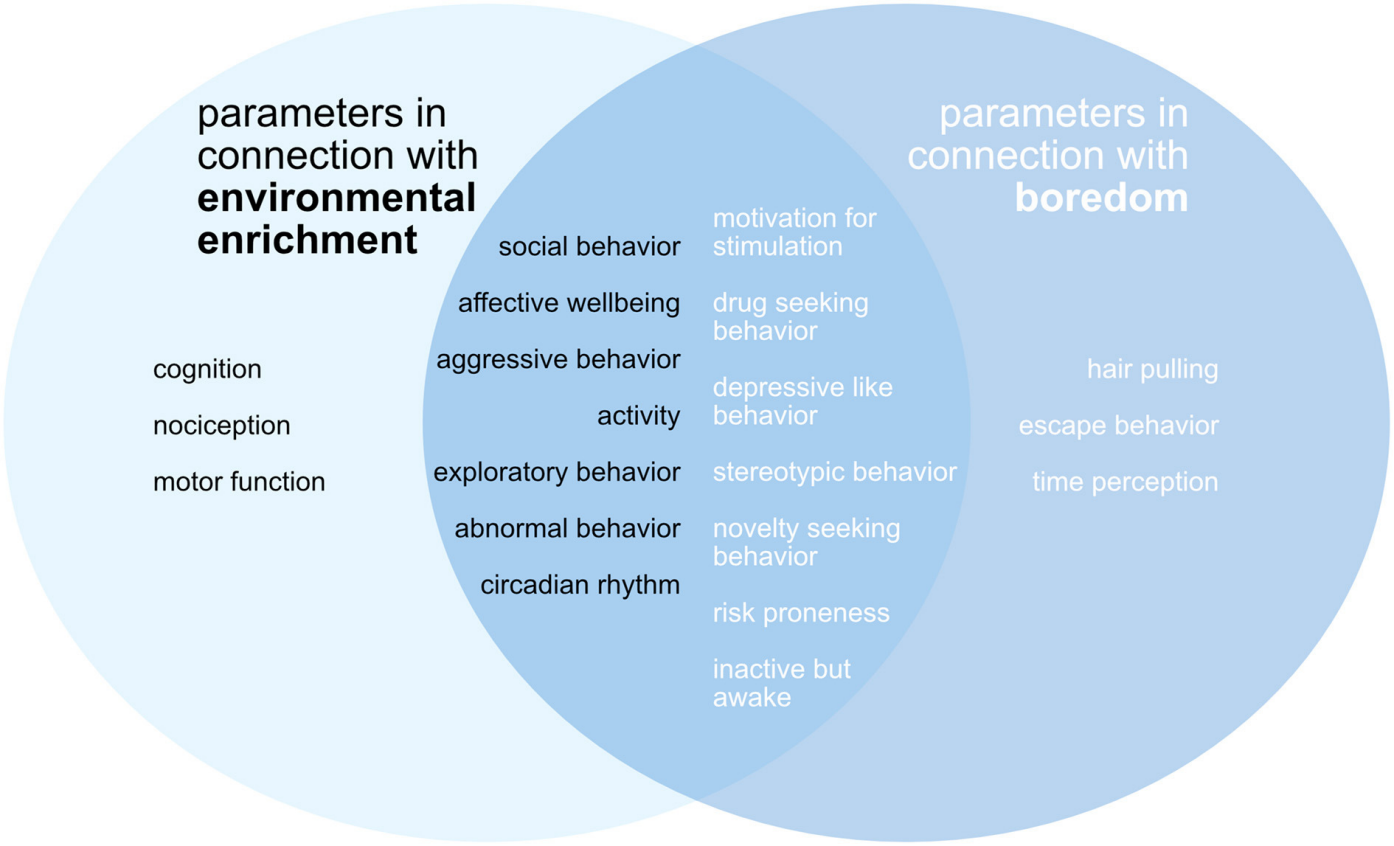
Scheme of the assessed outcome parameters with reference to enriched environment and boredom. Left in black letters: parameters in connection with environmental enrichment. Right in white letters: parameters in connection with boredom. There is a considerable overlap between the categorized parameters in the examined publications and the parameters associated with boredom.

Figure 2 shows the parameters that were examined in the context of environmental enrichment. The figure also shows the parameters that were defined as indicators of boredom and explicitly searched for in the publications. There is a large overlap between the factors examined in the studies and the boredom-related parameters.

### Increasing number of publications about home cage enrichment

**Figure 3:**
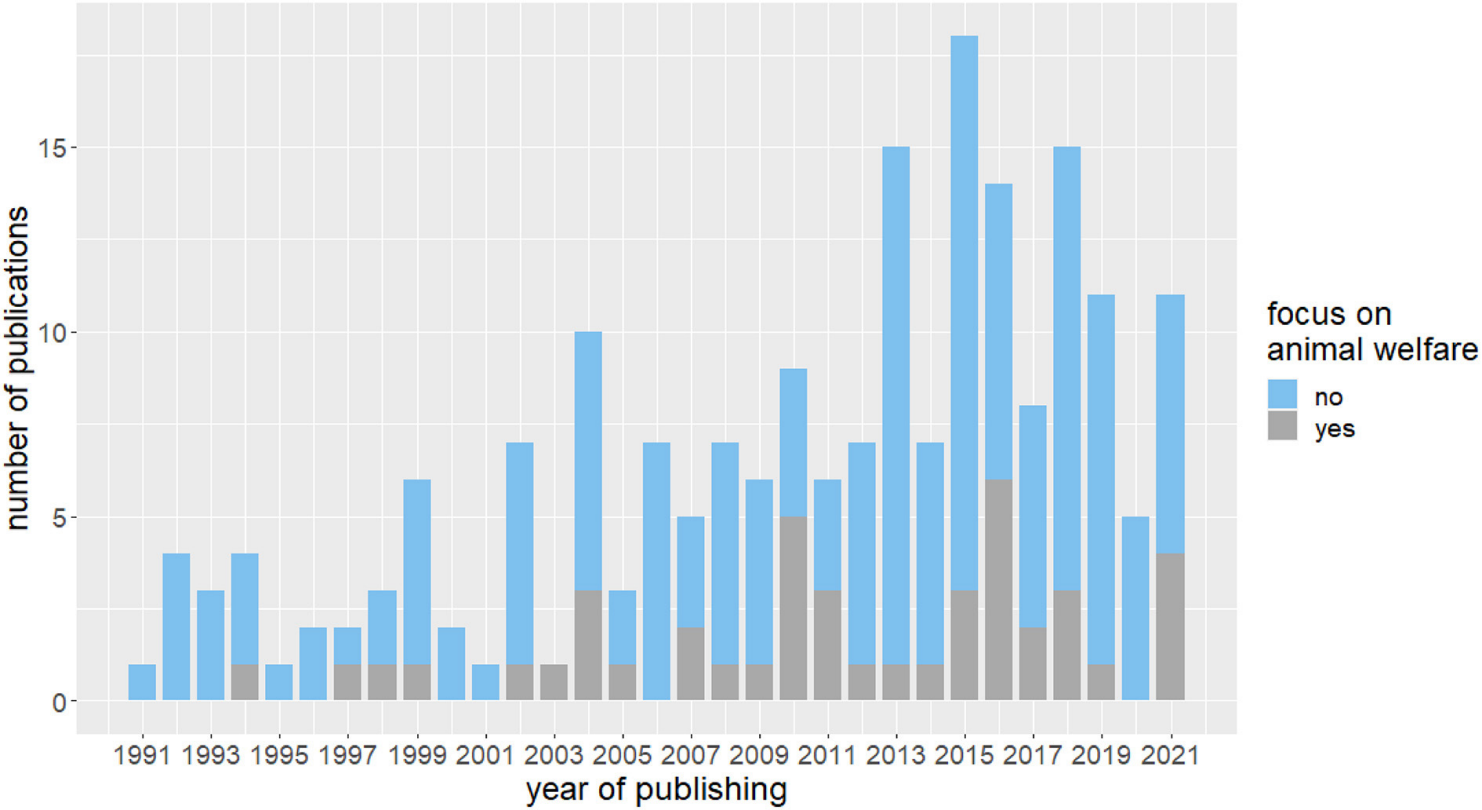
Absolute number of included publications in the years 1991 to 2022. Indicated is the number of publications with and without explicit focus on animal welfare in the publications.

The number of studies examining the effects of enriched housing on mouse and rat behavior has steadily increased, particularly over the past decade, with peaks in 2013, 2015, and 2018 (Figure 3). In 2022, three papers were included in the parameter extraction with one of them focusing on animal welfare. All studies that explicitly aimed to improve the housing conditions of laboratory animals and thus were dedicated to refining animal experiments were categorized as “Focus on animal welfare”. Although the absolute number of publications with a focus on animal welfare was slightly increasing over time, its overall proportion is still low.

### Results on reviewed methods and experimental designs

**Figure 4:**
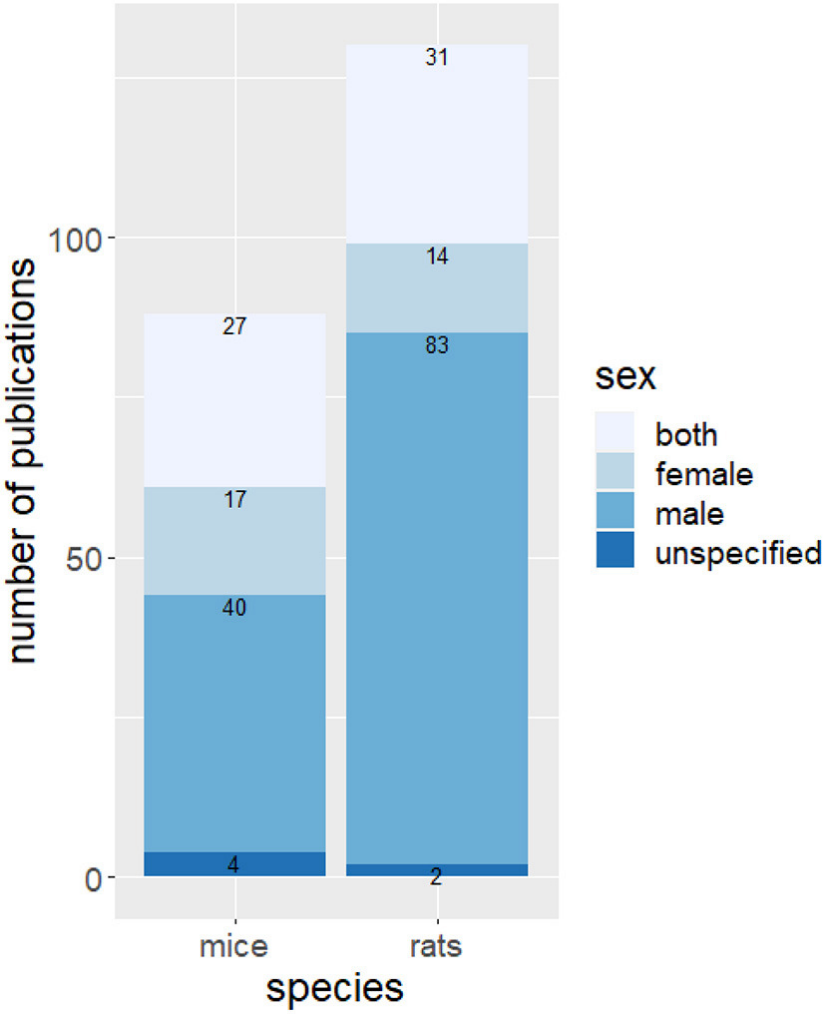
Number of publications using rats and mice and sex bias. Indicated is the absolute number of publications with the specified species and sexes.

Rats have been used more frequently than mice to study the effects of housing conditions on behavior and for both species, mainly males were examined (Figure 4). The most frequently used rat strain was Sprague-Dawley (48 studies) followed by Wistar (44 studies). Twenty-two studies housed Long-Evans rats as experimental animals. Eighteen different strains of mice were studied in the context of environmental enrichment. The most used strains were C57BL/6 (39 studies), BALB/C (13 references) and CD-1 mice (11 references).

The enrichment applied in the examined studies was divided into three categories. “Social enrichment” was defined as being housed in a group or provided with a cage partner. When additional space by increasing the home cage size was used to provide enrichment, the category ‘size enrichment' was indicated. The “object enrichment” category was assigned when the environment was changed by the introduction of objects of any kind (toys, climbing opportunities, structural elements).

Most studies used a combination of all three types of enrichment in their experiments (104 studies). This was followed by a combination of object enrichment and size enrichment of the home cage (55 studies). Social enrichment alone (6 studies), enrichment of home cage space alone (3 studies) and the combination of social and spatial enrichment (3 studies) were the least used types of enrichment. Three studies used environmental enrichment in their experiments but did not mention the type.

### A stimulating environment is essential for the development of natural behavior and animal welfare

**Figure 5:**
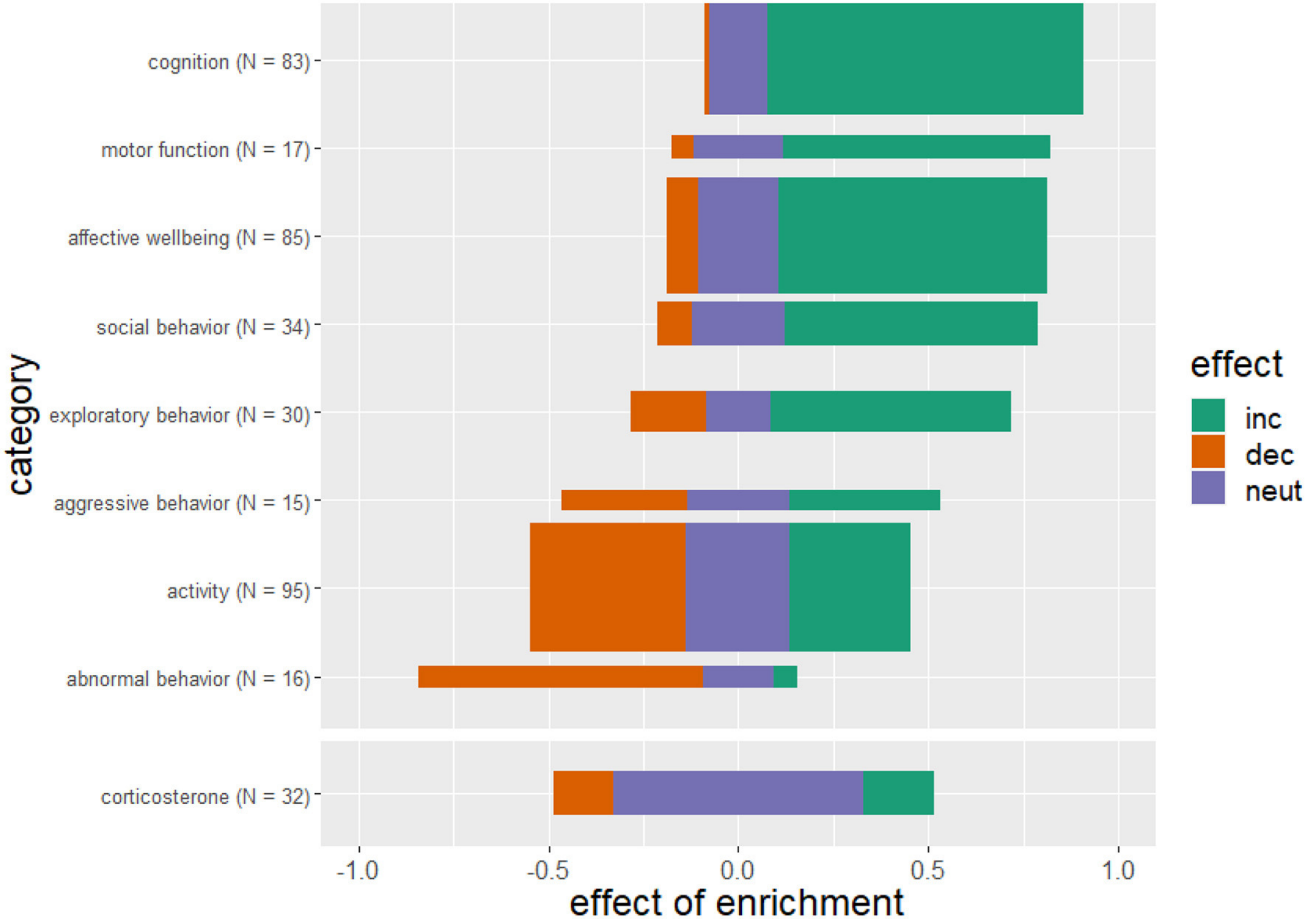
Effects of enriched housing on behavioral outcome and corticosterone level. Bars represent the studies that reported an increase (inc), a decrease (dec) or no change (neut) in the parameters of the corresponding category. Values indicate the observed effect of the enrichment as a decimal number. The thickness of the bars reflects the number of investigated studies for this category. The sum of references per category is greater than *N* = 218 studies because some studies examined more than one outcome parameter.

Providing animals with an enriched environment substantially improves cognitive skills. Motor function, social behaviors and affective state were positively affected, and abnormal behaviors were considerably decreased compared to conventional or barren housed animals, also indicating a positive protective effect. The effects of enrichment on the categories aggressive behavior and activity though remain inconclusive. There is no clear tendency for stress hormones to increase or decrease in relation to housing conditions (Figure 5).

### Enriched housing promotes well–being in mice and rats, and regardless of sex and age

**Figure 6:**
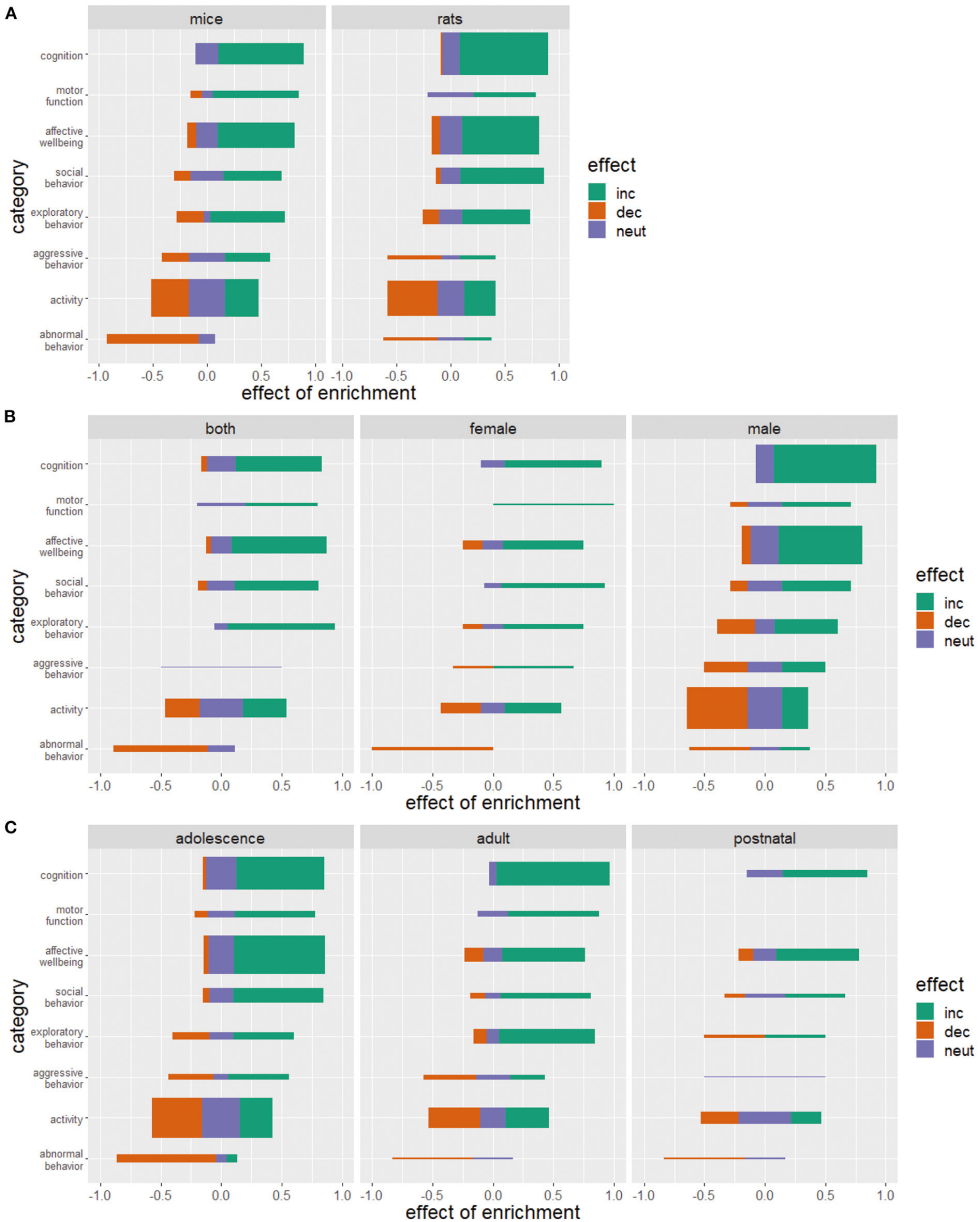
Effects of enriched housing on mice and rats **(A)**, in relation to sex **(B)**, and age **(C)**. Bars represent the studies that reported an increase (inc), a decrease (dec) or no change (neut) in the parameters of the corresponding category. Values indicate the observed effect of the enrichment as a decimal number. The thickness of the bars reflects the number of investigated studies for this category. **(C)** Animals were considered postnatal at the age of 0–21 days, adolescent at the age of 21–60 days, adult at the age of 60–750 days and post reproductive at the age of more than 750 days.

The reported effects of environmental enrichment on animal welfare are largely independent of the animal species compared in this study. Mice and rats benefit similarly from enrichment of their living environment (Figure 6A).

Most of the studies examined were performed on males (123 studies, Figure 6B). Fifty-eight studies examined both sexes whereas only 31 studies did experiments on female animals. Enrichment increases cognition, social behavior and motor function and decreases abnormal behavior in females and males, with these effects being more pronounced in females. Regardless of sex, a similar number of studies reported an impairment, a reduction, or no effect on activity. Exploration and aggressive behavior in females increased with the provision of enrichment. Eight studies examined the effect of enrichment on aggressive behavior in male animals. In four of these studies, an increase in aggressive behavior was observed.

Most of the studies reviewed were conducted with adolescent animals (117 studies, Figure 6C). Seventy studies used adult animals and 29 studies used postnatal animals. Two studies used post-reproductive animals. Apart from this discrepancy in the use of animals of different ages, the effects of enrichment on cognition, affective well-being, social behavior, and the development of abnormal behavior proved generally positive for all age groups. Motor function was positively affected by enrichment but data in postnatal and adult animals are lacking here as well as in post-reproductive animals. Ambiguous results of the effect of enrichment on aggressive behavior, exploratory behavior, and activity with an increase, decrease as well as a neutral or no effect could be detected.

### The longer the period of housing in an enriched environment, the higher the benefit to welfare

**Figure 7:**
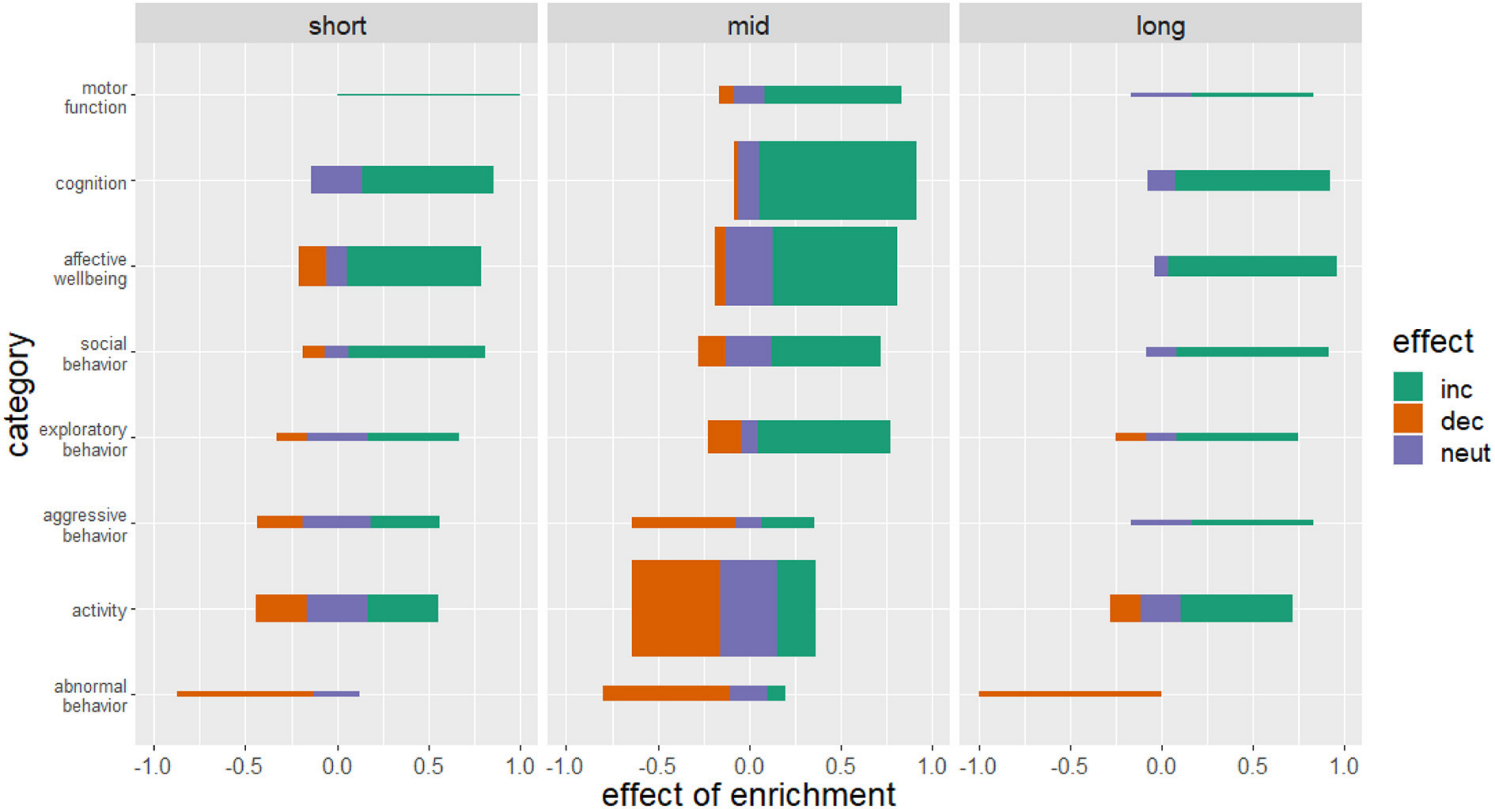
Effects of enriched housing in relation to housing duration. Bars represent the studies that reported an increase (inc), a decrease (dec) or no change (neut) in the parameters of the corresponding category. Values indicate the observed effect of the enrichment as a decimal number. The thickness of the bars reflects the number of investigated studies for this category. Duration of enriched housing was classified in short housing duration of 0–30 days, mid housing duration of 30–90 days and long-term housing duration of more than 90 days.

Most of the included studies applied a medium housing period (30–90 days, 124 studies). The most beneficial effect of enrichment was obtained with a long housing duration (more than 90 days, 33 studies) but all durations could improve motor function, cognition and affective well–being and exert a protective effect against the development of abnormal behavior (Figure 7). The effect of enrichment duration on aggressive behavior and activity remained inconclusive with a tendency to an increase in aggressive behavior and activity in a long-term provision of enrichment.

## Discussion

Environmental enrichment has been a popular research topic for some time, not excessively but continually researched. Neuroscience research has provided some fundamental results in this field, elucidating the close relationship of animal housing conditions on the structure and function of the central nervous system. Most published studies use enrichment as an intervention in animal models of various diseases, including stroke (127, 128), traumatic brain injury (129), and Alzheimer's disease (10). Although this is a highly exciting field of research, these studies were deliberately not included in this systematic review. This systematic review instead focuses on enriched environment as a means of preventing boredom-like symptoms and improving the welfare of laboratory animals.

While research activity on enriched environments has increased steadily over the years, only a small fraction of the investigated studies dealt specifically with animal welfare. This is perhaps not surprising, since there are various definitions of animal welfare (130), and no consensus on how to improve it. However, our data show that the proportion of studies with a specific focus on improving the living conditions of laboratory animals in enrichment research is slightly rising. As animal welfare research gains increasing recognition as an established research discipline, the number of research papers in the field will likely continue to grow. For example, recent research shows that tunnel handling can improve physiological well-being and often the handling tunnel is used as an additional enrichment item (131).

Our analysis shows that rats are used more frequently than mice in enrichment research and that different strains of both species are used. Nevertheless, rats and mice benefit similarly from an enriched living environment and there is no evidence that housing conditions affect the welfare of strains differently. Females are underrepresented in studies with mice and even more so in studies with rats. Among the studies using mice, 31% reported the use of both sexes, 46% the use of male, and 19% the use of female mice. In the rat studies, 24% used both sexes, 64% used male, and only 11% used female rats. A similar bias toward the use of male subjects has been found in preclinical animal research (132). The underrepresentation of female subjects in animal research is based on the belief that females are more variable than males due to their estrous cycle. However, for most applications including behavioral measures, female rodents display no more variation than males do; and female estrus cycles therefore need not necessarily be given special consideration (133). The underrepresentation of females in animal research is still pervasive, and the scientific understanding of female biology is compromised by these persistent disparities. To address the inadequate inclusion of female animals, the US National Institutes of Health has implemented policies in 2014 that require applicants to indicate their plans for a balance of males and females in preclinical studies in all future applications, unless sex inclusion is not warranted due to strictly defined exceptions (134). The bias toward male subjects in animal research is receiving additional attention due to a plausible implication in the much-discussed translational crisis. Less consideration has so far been devoted to the obvious ethical implications of this sex imbalance. Since no fewer females than males are born in breeding facilities for laboratory animals, the question inevitably arises as to what happens to the “surplus” females (130).

Age is another important experimental factor in animal research that is often inadequately considered in experimental design and poorly reported in publications. Animals used in the examined enrichment studies tend to be young. In most of the studies, the housing phase in the enriched cages started at 0–4 weeks of age. In the behavioral tests, many of the animals were then tested at 6–14 weeks of age. This corresponds to the average age of 8–12 weeks at which laboratory animals are usually used in animal research (135). At this age, many developmental processes are not yet complete. It is therefore important to note that age-related physiological changes can have a major influence on experimental outcomes.

The positive effects of a diversified housing on physical, cognitive, and affective health of laboratory animals have been demonstrated by numerous publications analyzed in this review. Motor function, cognition, affective well–being, and social behavior benefited most from enriched housing. A reduction in abnormal behavior was also frequently reported with enriched housing. The effect of enrichment on activity remains inconclusive. One possible reason for the ambiguous results on the activity parameter is the broad definition of the parameter, which might limit the interpretability. Another reason could be the observed decrease in abnormal behaviors (stereotypies) due to housing in an enriched environment, which are usually accompanied by a significant level of activity. Since an enriched environment is often associated with more space and/or the provision of a running wheel, animals in these housing conditions clearly have more opportunity for physical activity than animals in confined housing. Mice housed in enriched cage systems outperformed conventionally housed animals on the rotarod, indicating that enrichment stimulates motor coordination and presumably fitness, even when no running wheel or disc is provided (136). Numerous studies on animals and humans have evidenced the beneficial influence of physical activity on the musculoskeletal system (137, 138). It is therefore a reasonable assumption that keeping laboratory animals in confined cages can harm the bone structure and musculature of laboratory animals.

Interestingly, we did not detect a clear increase or decrease in glucocorticoid stress hormones associated with housing conditions. In a recent review, however, it was suggested that conventional laboratory housing was found to be associated with chronic stress (7). Instead of a chronic increase in stress hormones, we suggest that conventional housing may rather reduce the capacity of the stress axis to cope with environmental challenges and that the health impairments result from constant under-stimulation. This would be in line with the proposed non-linear relation of stress and welfare as proposed by Korte et al. (139). However, it should be noted that the determination of stress hormones in the included publications was very heterogeneous in terms of sample source, number, and timing and that these parameters were not assessed. This evaluation was not a central topic of this work, and measurement of glucocorticoid stress hormones was not a part of the search strategy. However, our preliminary data suggest that a more thorough analysis of this parameter may be warranted.

The effects of a stimulus-rich environment on cognition and affective well–being are well–documented and there is accumulating evidence for potential underlying brain structures and neurophysiological mechanisms. These extend from brain region volume and morphology to neuron complexity and excitability, adult neurogenesis, synaptic plasticity, and a plethora of molecular responses including gene-environment interactions, inflammation, and trophic factors (140–143). Many of these effects are likely linked to the increased physical activity associated with an enriched housing. However, there are processes that are directly attributable to the stimulative elements of enrichment. These include the successful differentiation and long-term survival of newly formed neurons during neurogenesis, processes that can be clearly distinguished from the proliferation of neural cells, which in turn is facilitated in particular by physical activity (144).

In the studies reviewed, a variety of housing, bedding, and nesting materials, as well as various items or any combination thereof, were used as enrichment. It is worth noting that pre-build shelters can have different effects than providing material for building their own nests (145). Historically, all additions to housing cages were considered enrichment. In this way, “enrichment” became an umbrella term for a variety of shelters, bedding and nesting materials, and miscellaneous items, or any combination thereof, and lacked a general theoretical framework for what should be considered enrichment (4). This is also reflected in the studies reviewed. In most publications, a combination of social, object and spatial enrichment was used (Supplementary Table 2). Because of the widespread simultaneous use of all types of enrichment, there is no clear consensus on which form is most effective in preventing housing-specific behavioral disorders.

### Enriched environment alleviates boredom-like symptoms in laboratory animals

Some of the outcomes extracted in this review may be directly related to boredom in laboratory animals. These included abnormal behaviors like stereotypic, hyperactivity, and inactive-but-awake behavior, as well as novelty-seeking, drug-seeking, and depressive like behavior. Thirty-three publications dealt with novelty-seeking behavior in the broadest sense (Table 1). This parameter is often investigated with the open field test or elevated plus maze, but also by observing the behavior or activity in newly presented home cages. While novelty seeking is assumed to be an indicator of boredom, the measurement of novelty seeking is often linked to activity and exploration in a range of different tests. This makes it difficult to clearly attribute the results of tests classified as novelty seeking in terms of boredom. Therefore, there is no unequivocal effect of environmental enrichment on novelty seeking behavior.

Twenty-three of the included publications investigated depressive like behavior in connection with environmental enrichment. This was mostly done with the forced swim test and tail suspension test. Fifteen studies (65%) describe a decrease and four (17, 4%) an increase in symptomatology in animals housed in an enriched environment. The 10 most recent studies published since 2014 uniformly show a decrease in depressive-like behavior in animals housed in enriched environments.

Twenty-four publications were identified as studies on drug-seeking behavior. Here, a consistently positive effect of environmental enrichment was reported.

Sixteen studies examined stereotypic behavior in mice and rats were. There was an overall decrease of stereotypic behavior under enriched housing conditions. Although the occurrence of stereotypic behavior appears to be a multifactorial event in animals (6, 15), it can be observed more frequently under barren restrictive housing conditions and has been shown to be reduced by the use of enrichment in zoo animals (146). Burn (21) argued that stereotypic behaviors increase under monotonous situations and identified abnormal repetitive behaviors as a potential measurable boredom parameter in captive animals.

Very poorly represented are the boredom parameters motivation for stimulation, inactive but awake and risk proneness with 12 publications in total. These characteristics, which closely relate to human boredom, are also influenced by environmental enrichment. Motivation for stimulation is a parameter that has been reported to be both increased and decreased by an enriched environment. This parameter is usually derived from the activity behavior of the animals and determined by a variety of tests that lead to inconclusive results. Awake inactivity was reduced by enriched environment in every included publication. Two studies found increased, and one found unchanged risk proneness, in animals living in an enriched environment. However, with only three publications related to risk proneness in our body of literature, this statement should be viewed with caution.

Escape behavior, hair pulling, or a possible shift of time perception were not examined by any publication. Overall, it must be noted that in only a few cases boredom was specifically mentioned at all.

### Methodological considerations

Although boredom is resonant in many enrichment studies, it is almost never directly examined and rarely mentioned at all. Due to limited data availability, conducting a meta-analysis on this particular topic is not feasible. Nevertheless, to approach the topic, we developed a systematic review in which we investigate the effect of animal husbandry on the welfare of laboratory animals and assign some of the extracted welfare parameters to typical symptoms of boredom. Since boredom and animal welfare are multifaceted conditions, this work is not based on the investigation of a single outcome, as considered in the classical PICO scheme but examines a set of parameters related to welfare and potentially boredom of laboratory animals.

The evaluation of the compliance with the established scientific quality criteria in the examined studies revealed a common lack of reported blinding. The percentage of about 25% of studies reporting blinding seems to be relatively low especially compared to preclinical biomedical studies (147) and also compared with a recent meta-analysis of the effects of housing on mortality in animal models of disease (7). One possible reason for this could be that behavioral studies are increasingly automated and/or conducted in the home cage without any required intervention with a (blinded) experimenter. In the case of behavioral observations in the (enriched) home cage, blinding of the observer is difficult to implement; in the case of automated behavioral analyses, it may not be necessary. This was not explored in this work; however, a systematic review of the use of automated and home cage-based systems for behavior analysis would be intriguing.

Although the study protocol was determined a priori, the protocol of this systematic review was not pre-registered. While this was not done in this work, it should be emphasized here that prospective registration of systematic reviews and meta-analyses reduces the potential for bias and fosters transparency (148).

## Conclusion

Our findings show that a stimulating environment can be considered essential for the development of natural behavior and animal welfare of research rodents. Although boredom is almost never studied directly and rarely mentioned, this theme clearly resonates in many studies of the effects of improved housing conditions. Chronic boredom as a consequence of living in a barren and confined environment can pose a health risk to laboratory animals, limiting their validity as model organisms for biomedical research. A stimulating living environment sustains the well–being of laboratory rats and mice alike, regardless of age and sex. Although a longer period of housing might be more beneficial, even a short period in a stimulating environment improves essential parameters of animal welfare. Providing animals with adequate space, social contact, and a stimulating environment should not be considered a luxury or a treatment, but a necessity to ensure mental and physical health and a foundation for the expression of natural behaviors.

## Data availability statement

The original contributions presented in the study are included in the article/Supplementary material, further inquiries can be directed to the corresponding author.

## Author contributions

PM, UH, DB, LL, and KD: developed the study concept and design. PM, UH, CF-T, CH, KH, PK, JM, JR, JW, LL, and KD: were involved in data acquisition and analysis. PM, UH, LL, and KD: drafted the manuscript and figures. All authors contributed to the article and approved the submitted version.

## Funding

This work was funded by the German Federal Institute for Risk Assessment.

## Conflict of interest

The authors declare that the research was conducted in the absence of any commercial or financial relationships that could be construed as a potential conflict of interest.

## Publisher's note

All claims expressed in this article are solely those of the authors and do not necessarily represent those of their affiliated organizations, or those of the publisher, the editors and the reviewers. Any product that may be evaluated in this article, or claim that may be made by its manufacturer, is not guaranteed or endorsed by the publisher.
