# Supplementary material for: Bored at home?—A systematic review on the effect of environmental enrichment on the welfare of laboratory rats and mice
Source: Front Vet Sci. 2022 Aug 18;9:899219. doi: 10.3389/fvets.2022.899219 (PMC9435384; doi:10.3389/fvets.2022.899219)
Supplement: Supplementary file 1 [file Table_1.DOCX]

Table S1. Categorized parameters, their description, and the behavioral tests used in the included studies. The behavioral tests and the descriptions are assigned to the respective category. The number in parentheses indicates multiple occurrences of tests in the examined studies. Behavioral tests may be listed multiple times if studies investigated more than one parameter with the same test.

| **categorized parameter** | **description** | **behavioral test** |
| --- | --- | --- |
| social behavior | all measurable individual behaviors between two or more individuals. | behavioral observation (home cage) via video/live (n=17), social exploratory behavior/open field (n=12), social interaction test (n=3), mate choice test (n=3), 3-chamber sociability test (n=2), intruder test (n=2), social dominance test (n=2), passive avoidance test, runway test, step down inhibitory avoidance (maternal), social interaction and memory test, competitive food foraging test, maternal defense test, pup-retrieval test, predator odor task |
| cognition | all measurable processes of learning, memory, and attention | mazes (n=42, radial arm, Morris water, T, Y, Barnes), object recognition task (n=11), operant chamber/discrimination task (n=5), olfactory temporal order discrimination (OTOD) task, delayed match-to-place (DMTP) and delayed spatial win-shift (DSWSh) discrimination tasks, conditioned taste aversion, two-choice discrimination task, spatial reference memory task, passive avoidance task, simple visual discrimination task, sucrose reinforcement test, visual water task, two-choice guessing task, Hebb–Williams closed field test, discriminative learning task, social interaction and memory test, forced-choice delayed-alternations task, Pavlovian conditioned freezing & foreground contextual conditioning, test apparatus with touchscreen and locomotor monitoring, passive avoidance task, simple visual discrimination task, vestibular oculomotor learning, variable interval delayed alternation, contextual fear conditioning, conditioned place preference |
| affective wellbeing | all measurable mood states (such as anxiety or depression) that are not caused by a single stimulus but are the result of an accumulation of experiences. | elevated mazes (n=36, plus, zero), open field (n=34), forced swim test (n=15), behavioral observation (home cage) via video/live (n=12), light-dark test (n=7), sucrose preference test (n=5), tail suspension test (n=3), water escape task, novel object task, predator odor task, water escape exposure, brain-stimulation reward test, free exploration test, electric shock, operant conditioning chambers, free-exploratory paradigm, novel cage stressor test, judgment bias, operant chambers, anticipatory reaction to handling, mouth gag cooperation test, human approach test, predator exposure, prepulse inhibition, cognitive bias test, operant nosepoke task, food neophobia, hole-board test, foot-shock test, tail withdrawal test, tail flick test, tone response test, water escape exposure, corridor field task, choice drinking test |
| aggressive behavior | all measurable antagonistic behaviors between two or more individuals | behavioral observation (home cage) via video/live (n=8), intruder test (n=4), group test (n=2), pup retrieval test, maternal aggression test, maternal defense test, maternal aggression test, social interaction test, competitive food foraging test, novel cage stressor test, social approach test, social dominance test, social interaction test |
| activity | all measurable behaviors that provide information about physical activity | open field (n=53), mazes (n=43, elevated plus, radial arm, Morris water, T, Y, Barnes), behavioral observation (home cage) via video/live (n=25), automated activity measurement (n=21), object exploration tests (n=12), conditioned place/stimulus preference tests, nose poke exploration, novel cage stressor test, spontaneous alternation test, corridor field task, running wheel |
| abnormal behavior | all measurable behaviors that differ from typical species-specific behaviors (such as stereotypic behavior or barbering). | behavioral observation (home cage) via video/live (n=11), automated activity measurement (n=3), open field behavior, spontaneous stereotypies, digging problem solving task |
| nociception | all measurable behaviors related to the perception of pain | tail flick test (n=2), hot plate test (n=2), formalin test, paw pressure test |
| motor function | all measurable behaviors related to motor function and motor coordination | rotarod (n=4), grip-strength test (n=2), staircase test, ladder walking test, string suspension test, skilled reaching task, motor activity units, inclined plane, motility box, beam walking test, hind limb gait test |
| circadian rhythm | all measurable behaviors related to the species-specific circadian rhythm | video analysis, exercise activity running wheel |
| exploratory behavior | all measurable behaviors directed toward the acquisition of information about the environment. | open field (n=19), mazes (n=13, elevated plus, Morris water, Y, 0), behavioral observation (home cage) via video/live (n=6), object recognition test (n=6), hole board (n=3), dark preference test |
