# Supplementary material for: Bored at home?—A systematic review on the effect of environmental enrichment on the welfare of laboratory rats and mice
Source: Front Vet Sci. 2022 Aug 18;9:899219. doi: 10.3389/fvets.2022.899219 (PMC9435384; doi:10.3389/fvets.2022.899219)
Supplement: Supplementary file 2 [file Table_2.DOCX]

Table S2. Overview of the use of different types of enrichment and their effects on behavioral outcome measures. In bold is the number of studies that reported an increase in the respective parameter. The number after the slash indicates the total number of cases in which environmental enrichment was used to influence the respective behavioral parameter.

|  | **Type of enrichment** | | | | | | |
| --- | --- | --- | --- | --- | --- | --- | --- |
|  | **social** | **object** | **space** | **social & object** | **social & space** | **object & space** | **social, object & space** |
| **exploratory behavior** | **0**/ 0 | **5**/ 6 | **0**/ 0 | **2**/ 3 | **0**/ 0 | **7**/ 10 | **9**/ 16 |
| **motor function** | **0** / 0 | **0** / 0 | **0** / 0 | **2** / 2 | **0** / 0 | **5** / 8 | **4** / 6 |
| **abnormal behavior** | **0** / 0 | **0** / 6 | **0** / 1 | **0** / 1 | **0** / 0 | **0** / 5 | **1** / 4 |
| **activity** | 2 / 4 | 6 / 20 | **1** / 2 | **4** / 6 | **0** / 1 | **10** / 26 | **7** / 40 |
| **aggressive behavior** | **0** / 0 | **2** / 5 | **0** / 0 | **0** / 1 | **0** / 0 | **2** / 6 | **2** / 5 |
| **affective wellbeing** | **4** / 4 | 7 / 11 | **1** / 1 | **5** / 6 | **0** / 2 | **20** / 24 | **26** / 40 |
| **cognition** | **0** / 0 | 4 / 6 | **0** / 0 | **3** / 3 | **0** / 0 | 19 / 23 | **44** / 54 |
| **social behavior** | **1** / 1 | **2** / 3 | **0** / 0 | **1** / 1 | **0** / 0 | **6** / 12 | **13** / 17 |
| **Total number of enrichment type used** | **9** | **57** | **4** | **23** | **3** | **117** | **185** |
