# Supplementary material for: Bored at home?—A systematic review on the effect of environmental enrichment on the welfare of laboratory rats and mice
Source: Front Vet Sci. 2022 Aug 18;9:899219. doi: 10.3389/fvets.2022.899219 (PMC9435384; doi:10.3389/fvets.2022.899219)
Supplement: Supplementary file 3 [file Data_Sheet_1.docx]

**References included in the review**

1. Abou-Ismail, U. A., Burman, O. H., Nicol, C. J. & Mendl, M. The effects of enhancing cage complexity on the behaviour and welfare of laboratory rats. *Behav Process.* **85**, 172–180 (2010).

2. Abramov, U., Puussaar, T., Raud, S., Kurrikoff, K. & Vasar, E. Behavioural differences between C57BL/6 and 129S6/SvEv strains are reinforced by environmental enrichment. *Neurosci. Lett.* **443**, 223–227 (2008).

3. Abramov, U. *et al.* Different housing conditions alter the behavioural phenotype of CCK(2) receptor-deficient mice. *Behav. Brain Res.* **193**, 108–116 (2008).

4. Adcock, A. *et al.* Where are you from? Female mice raised in enriched or conventional cages differ socially, and can be discriminated by other mice. *Behav. Brain Res.* **400**, (2021).

5. Akillioglu, K., Yilmaz, M. B., Boga, A., Binokay, S. & Kocaturk-Sel, S. Environmental enrichment does not reverse the effects of maternal deprivation on NMDAR and Balb/c mice behaviors. *Brain Res.* **1624**, 479–488 (2015).

6. Altun, M., Bergman, E., Edström, E., Johnson, H. & Ulfhake, B. Behavioral impairments of the aging rat. *Physiol Behav* **92**, 911–923 (2007).

7. Augustsson, H., Lindberg, L., Hoglund, A. U. & Dahlborn, K. Human-animal interactions and animal welfare in conventionally and pen-housed rats. *Lab. Anim.* **36**, 271–281 (2002).

8. Aujnarain, A. B., Luo, O. D., Taylor, N., Lai, J. K. Y. & Foster, J. A. Effects of exercise and enrichment on behaviour in CD-1 mice. *Behav. Brain Res.* **342**, 43–50 (2018).

9. Azevedo de Meneses, J., Junqueira Lopes, C. A., Coca Velarde, L. G. & Teles Boaventura, G. Behavioral analysis of Wistar rats fed with a flaxseed based diet added to an environmental enrichment. *Nutr. Hosp.* **26**, 716–721 (2011).

10. Badowska, D. M., Brzózka, M. M., Chowdhury, A., Malzahn, D. & Rossner, M. J. Data calibration and reduction allows to visualize behavioural profiles of psychosocial influences in mice towards clinical domains. *Eur Arch Psychiatry Clin Neurosci* **265**, 483–496 (2015).

11. Bahi, A. Environmental enrichment reduces chronic psychosocial stress-induced anxiety and ethanol-related behaviors in mice. *Prog. Neuro-Psychopharmacology Biol. Psychiatry* **77**, 65–74 (2017).

12. Bailoo, J. D. *et al.* Effects of Cage Enrichment on Behavior, Welfare and Outcome Variability in Female Mice. *Front. Behav. Neurosci.* **12**, (2018).

13. Balietti, M., Pugliese, A., Fabbietti, P., Di Rosa, M. & Conti, F. Aged rats with different performances at environmental enrichment onset display different modulation of habituation and aversive memory. *Neurobiol. Learn. Mem.* **161**, 83–91 (2019).

14. Baraldi, T. *et al.* Cognitive stimulation during lifetime and in the aged phase improved spatial memory, and altered neuroplasticity and cholinergic markers of mice. *Exp Gerontol* **48**, 831–838 (2013).

15. Barbelivien, A. *et al.* Environmental enrichment increases responding to contextual cues but decreases overall conditioned fear in the rat. *Behav Brain Res* **169**, 231–238 (2006).

16. Bardi, M. *et al.* Paper or Plastic? Exploring the Effects of Natural Enrichment on Behavioural and Neuroendocrine Responses in Long-Evans Rats. *J. Neuroendocrinol.* **28**, (2016).

17. Barker, T. H., Howarth, G. S. & Whittaker, A. L. The effects of metabolic cage housing and sex on cognitive bias expression in rats. *Appl. Anim. Behav. Sci.* **177**, 70–76 (2016).

18. Bechara, R. G. & Kelly, A. M. Exercise improves object recognition memory and induces BDNF expression and cell proliferation in cognitively enriched rats. *Behav. Brain Res.* **245**, 96–100 (2013).

19. Bechard, A. R., Cacodcar, N., King, M. A. & Lewis, M. H. How does environmental enrichment reduce repetitive motor behaviors? Neuronal activation and dendritic morphology in the indirect basal ganglia pathway of a mouse model. *Behav. Brain Res.* **299**, 122–131 (2016).

20. Bechard, A. R., Bliznyuk, N. & Lewis, M. H. The development of repetitive motor behaviors in deer mice: Effects of environmental enrichment, repeated testing, and differential mediation by indirect basal ganglia pathway activation. *Dev. Psychobiol.* **59**, 390–399 (2017).

21. Benaroya-Milshtein, N. *et al.* Environmental enrichment in mice decreases anxiety, attenuates stress responses and enhances natural killer cell activity. *Eur. J. Neurosci.* **20**, 1341–1347 (2004).

22. Bors, D. A. & Forrin, B. The effects of post-weaning environment, biological dam, and nursing dam on feeding neophobia, open field activity, and learning. *Can. J. Exp. Psychol.* **50**, 197–204 (1996).

23. Bourgeon, S., Xerri, C. & Coq, J. O. Abilities in tactile discrimination of textures in adult rats exposed to enriched or impoverished environments. *Behav Brain Res* **153**, 217–231 (2004).

24. Brancato, A., Castelli, V., Lavanco, G. & Cannizzaro, C. Environmental Enrichment During Adolescence Mitigates Cognitive Deficits and Alcohol Vulnerability due to Continuous and Intermittent Perinatal Alcohol Exposure in Adult Rats. *Front. Behav. Neurosci.* **14**, (2020).

25. Branchi, I. *et al.* Early social enrichment augments adult hippocampal BDNF levels and survival of BRDU-positive cells while increasing anxiety- and ‘depression’-like behavior. *J. Neurosci. Res.* **83**, 965–973 (2006).

26. Brenes, J. C., Padilla, M. & Fornaguera, J. A detailed analysis of open-field habituation and behavioral and neurochemical antidepressant-like effects in postweaning enriched rats. *Behav. Brain Res.* **197**, 125–137 (2009).

27. Brenes, J. C., Rodriguez, O. & Fornaguera, J. Differential effect of environment enrichment and social isolation on depressive-like behavior, spontaneous activity and serotonin and norepinephrine concentration in prefrontal cortex and ventral striatum. *Pharmacol. Biochem. Behav.* **89**, 85–93 (2008).

28. Brydges, N. M., Leach, M., Nicol, K., Wright, R. & Bateson, M. Environmental enrichment induces optimistic cognitive bias in rats. *Anim. Behav.* **81**, 169–175 (2011).

29. Cain, M. E., Green, T. A. & Bardo, M. T. Environmental enrichment decreases responding for visual novelty. *Behav. Processes* **73**, 360–366 (2006).

30. Callard, M. D., Bursten, S. N. & Price, E. O. Repetitive backflipping behaviour in captive roof rats (Rattus rattus) and the effects of cage enrichment. *Anim. Welf.* **9**, 139–152 (2000).

31. Cao, W. Y. *et al.* Early enriched environment induces an increased conversion of proBDNF to BDNF in the adult rat’s hippocampus. *Behav. Brain Res.* **265**, 76–83 (2014).

32. Cao, W. Y. *et al.* Role of early environmental enrichment on the social dominance tube test at adulthood in the rat. *Psychopharmacology (Berl).* **234**, 3321–3334 (2017).

33. Caporali, P. *et al.* Interaction does Count: A Cross-Fostering Study on Transgenerational Effects of Pre-reproductive Maternal Enrichment. *Front. Behav. Neurosci.* **9**, (2015).

34. Castelhano-Carlos, M. J., Baumans, V. & Sousa, N. PhenoWorld: addressing animal welfare in a new paradigm to house and assess rat behaviour. *Lab. Anim.* **51**, 36–43 (2017).

35. Chamizo, V. D., Rodríguez, C. A., Sánchez, J. & Mármol, F. Sex differences after environmental enrichment and physical exercise in rats when solving a navigation task. *Learn. Behav.* **44**, 227–238 (2016).

36. Chapillon, P., Manneche, C., Belzung, C. & Caston, J. Rearing environmental enrichment in two inbred strains of mice: 1. Effects on emotional reactivity. *Behav. Genet.* **29**, 41–46 (1999).

37. Chen, Y. *et al.* Environmental enrichment and chronic restraint stress in ICR mice: effects on prepulse inhibition of startle and Y-maze spatial recognition memory. *Behav Brain Res* **212**, 49–55 (2010).

38. Cheng, L., Wang, S. H., Jia, N., Xie, M. & Liao, X. M. Environmental stimulation influence the cognition of developing mice by inducing changes in oxidative and apoptosis status. *Brain Dev.* **36**, 51–56 (2014).

39. Clipperton-Allen, A. E. *et al.* Long-Term Provision of Environmental Resources Alters Behavior but not Physiology or Neuroanatomy of Male and Female BALB/c and C57BL/6 Mice. *J. Am. Assoc. Lab. Anim. Sci.* **54**, 718–730 (2015).

40. Coburn, J. F. & Tarte, R. D. The effect of rearing environments on the contrafreeloading phenomenon in rats. *J. Exp. Anal. Behav.* **26**, 289–294 (1976).

41. Cordier, J. M. *et al.* Postweaning Enriched Environment Enhances Cognitive Function and Brain-Derived Neurotrophic Factor Signaling in the Hippocampus in Maternally Separated Rats. *Neuroscience* **453**, 138–147 (2021).

42. Cortese, G. P., Olin, A., O’Riordan, K., Hullinger, R. & Burger, C. Environmental enrichment improves hippocampal function in aged rats by enhancing learning and memory, LTP, and mGluR5-Homer1c activity. *Neurobiol. Aging* **63**, 1–11 (2018).

43. Crawford, L. E. *et al.* Enriched environment exposure accelerates rodent driving skills. *Behav. Brain Res.* **378**, (2020).

44. Curley, J. P., Davidson, S., Bateson, P. & Champagne, F. A. Social enrichment during postnatal development induces transgenerational effects on emotional and reproductive behavior in mice. *Front. Behav. Neurosci.* **3**, (2009).

45. Cutuli, D. *et al.* Influence of Pre-reproductive Maternal Enrichment on Coping Response to Stress and Expression of c-Fos and Glucocorticoid Receptors in Adolescent Offspring. *Front. Behav. Neurosci.* **11**, (2017).

46. Cutuli, D. *et al.* Pre-reproductive maternal enrichment influences rat maternal care and offspring developmental trajectories: behavioral performances and neuroplasticity correlates. *Front. Behav. Neurosci.* **9**, (2015).

47. Cutuli, D. *et al.* Effects of pre-reproductive maternal enrichment on maternal care, offspring’s play behavior and oxytocinergic neurons. *Neuropharmacology* **145**, 99–113 (2019).

48. De Carvalho Chaves De Siqueira Mendes, F., Da Paixão, L. T. V. B., Diniz, C. W. P. & Sosthenes, M. C. K. Environmental impoverishment, aging, and reduction in mastication affect mouse innate repertoire to explore novel environments and to assess risk. *Front. Neurosci.* **13**, (2019).

49. De Carvalho, C. R., Pandolfo, P., Pamplona, F. A. & Takahashi, R. N. Environmental enrichment reduces the impact of novelty and motivational properties of ethanol in spontaneously hypertensive rats. *Behav. Brain Res.* **208**, 231–236 (2010).

50. De Oliveira, I. J. L., De Souza, V. V, Carvalho, A. C. P., Tomaz, C. & Da-Silva, S. L. Effects of ascorbic acid combined to environmental enrichment on anxiety and memory. *Psychol. Neurosci.* **12**, 363–378 (2019).

51. Dos Anjos-Garcia, T., Kanashiro, A., De Campos, A. C. & Coimbra, N. C. Environmental Enrichment Facilitates Anxiety in Conflict-Based Tests but Inhibits Predator Threat-Induced Defensive Behaviour in Male Mice. *Neuropsychobiology* (2022). doi:10.1159/000521184

52. Doulames, V., Lee, S. & Shea, T. B. Environmental enrichment and social interaction improve cognitive function and decrease reactive oxidative species in normal adult mice. *Int. J. Neurosci.* **124**, 369–376 (2014).

53. Eskola, S. & Kaliste-Korhonen, E. Aspen wood-wool is preferred as a resting place, but does not affect intracage fighting of male BALB/c and C57BL/6J mice. *Lab. Anim.* **33**, 108–121 (1999).

54. Falkenberg, T. *et al.* Increased expression of brain-derived neurotrophic factor mRNA in rat hippocampus is associated with improved spatial memory and enriched environment. *Neurosci. Lett.* **138**, 153–156 (1992).

55. Faraji, J. *et al.* Intergenerational sex-specific transmission of maternal social experience. *Sci. Rep.* **8**, (2018).

56. Franks, B., Champagne, F. A. & Higgins, E. T. How Enrichment Affects Exploration Trade-Offs in Rats: Implications for Welfare and Well-Being. *PLoS One* **8**, (2013).

57. Fureix, C. *et al.* Stereotypic behaviour in standard non-enriched cages is an alternative to depression-like responses in C57BL/6 mice. *Behav. Brain Res.* **305**, 186–190 (2016).

58. Garrido, P. *et al.* Differential effects of environmental enrichment and isolation housing on the hormonal and neurochemical responses to stress in the prefrontal cortex of the adult rat: Relationship to working and emotional memories. *J. Neural Transm.* **120**, 829–843 (2013).

59. Giacobbo, B. L. *et al.* Long-term environmental modifications affect BDNF concentrations in rat hippocampus, but not in serum. *Behav. Brain Res.* **372**, (2019).

60. Goes, T. C., Antunes, F. D. & Teixeira-Silva, F. Environmental enrichment for adult rats: Effects on trait and state anxiety. *Neurosci. Lett.* **584**, 93–96 (2015).

61. Greenough, W. T., Yuwiler, A. & Dollinger, M. Effects of posttrial eserine administration on learning in ‘enriched’ and ‘impoverished’ reared rats. *Behav. Biol.* **8**, 261–272 (1973).

62. Gregory, M. L. & Szurnlinski, K. K. Impoverished rearing impairs working memory and metabotropic glutamate receptor 5 expression. *Neuroreport* **19**, 239–243 (2008).

63. Gresack, J. E. & Frick, K. M. Environmental enrichment reduces the mnemonic and neural benefits of estrogen. *Neuroscience* **128**, 459–471 (2004).

64. Gresack, J. E., Kerr, K. M. & Frick, K. M. Short-term environmental enrichment decreases the mnemonic response to estrogen in young, but not aged, female mice. *Brain Res* **1160**, 91–101 (2007).

65. Grimm, J. W. *et al.* Brief Exposure to Novel or Enriched Environments Reduces Sucrose Cue-Reactivity and Consumption in Rats after 1 or 30 Days of Forced Abstinence from Self-Administration. *PLoS One* **8**, (2013).

66. Guan, S. Z. *et al.* The mechanism of enriched environment repairing the learning and memory impairment in offspring of prenatal stress by regulating the expression of activity-regulated cytoskeletal-associated and insulin-like growth factor-2 in hippocampus. *Environ. Health Prev. Med.* **26**, (2021).

67. Haemisch, A. & Gärtner, K. The cage design affects intermale aggression in small groups of male laboratory mice: strain specific consequences on social organization, and endocrine activations in two inbred strains (DBA/2J and CBA/J). *J. Exp. Anim. Sci.* **36**, 101–116 (1994).

68. Haemisch, A., Voss, T. & Gärtner, K. Effects of environmental enrichment on aggressive behavior, dominance hierarchies, and endocrine states in male DBA/2J mice. *Physiol. Behav.* **56**, 1041–1048 (1994).

69. Hannigan, J. H., Berman, R. F. & Zajac, C. S. Environmental enrichment and the behavioral effects of prenatal exposure to alcohol in rats. *Neurotoxicol Teratol* **15**, 261–266 (1993).

70. Hansen, S., Larsson, K., Carlsson, S. G. & Sourander, P. The development of sexual behavior in the rat: role of preadult nutrition and environmental conditions. *Dev Psychobiol* **11**, 51–61 (1978).

71. Hellemans, K. G. C., Benge, L. C. & Olmstead, M. C. Adolescent enrichment partially reverses the social isolation syndrome. *Dev. Brain Res.* **150**, 103–115 (2004).

72. Hendershott, T. R., Cronin, M. E., Langella, S., McGuinness, P. S. & Basu, A. C. Effects of environmental enrichment on anxiety-like behavior, sociability, sensory gating, and spatial learning in male and female C57BL/6J mice. *Behav. Brain Res.* **314**, 215–225 (2016).

73. Hobbiesiefken, U., Mieske, P., Lewejohann, L. & Diederich, K. Evaluation of different types of enrichment - their usage and effect on home cage behavior in female mice. *PLoS One* **16**, (2021).

74. Hoffmann, L. B. *et al.* Preconceptual paternal environmental stimulation alters behavioural phenotypes and adaptive responses intergenerationally in Swiss mice. *Physiol. Behav.* **223**, (2020).

75. Holm, L. & Ladewig, J. The effect of housing rats in a stimulus rich versus stimulus poor environment on preference measured by sigmoid double demand curves. *Appl. Anim. Behav. Sci.* **107**, 342–354 (2007).

76. Huzard, D., Mumby, D. G., Sandi, C., Poirier, G. L. & van der Kooij, M. A. The effects of extrinsic stress on somatic markers and behavior are dependent on animal housing conditions. *Physiol. Behav.* **151**, 238–245 (2015).

77. Inglis, I. R. Enriched sensory experience in adulthood increases subsequent exploratory behaviour in the rat. *Anim. Behav.* **23**, 932–940 (1975).

78. Irvine, G. I. & Abraham, W. C. Enriched environment exposure alters the input-output dynamics of synaptic transmission in area CA1 of freely moving rats. *Neurosci. Lett.* **391**, 32–37 (2005).

79. Iwata, E., Kikusui, T., Takeuchi, Y. & Mori, Y. Fostering and environmental enrichment ameliorate anxious behavior induced by early weaning in Balb/c mice. *Physiol. Behav.* **91**, 318–324 (2007).

80. Jaiswal, A. K., Upadhyay, S. N. & Bhattacharya, S. K. Effect of pyritinol, a cerebral protector, on learning and memory deficits induced by prenatal undernutrition and environmental impoverishment in young rats. *Indian J Exp Biol* **28**, 609–615 (1990).

81. Janus, C., Koperwas, J. S., Janus, M. & Roder, J. Rearing environment and radial maze exploration in mice. *Behav. Processes* **34**, 129–140 (1995).

82. Johnson, S. R., Patterson-Kane, E. G. & Niel, L. Foraging enrichment for laboratory rats. *Animal Welfare* **13**, 305–312 (2004).

83. Jones, M. A., Mason, G. & Pillay, N. Early environmental enrichment protects captive-born striped mice against the later development of stereotypic behaviour. *Appl. Anim. Behav. Sci.* **135**, 138–145 (2011).

84. Joseph, R. & Gallagher, R. E. Gender and early environmental influences on activity, overresponsiveness, and exploration. *Dev. Psychobiol.* **13**, 527–544 (1980).

85. Joshi, S. & Pillay, N. Personality predicts the responses to environmental enrichment at the group but not within-groups in stereotypic African striped mice, Rhabdomys dilectus. *Appl. Anim. Behav. Sci.* **182**, 44–52 (2016).

86. Joshi, S. & Pillay, N. Is wheel running a re-directed stereotypic behaviour in striped mice Rhabdomys dilectus? *Appl. Anim. Behav. Sci.* **204**, 113–121 (2018).

87. Kalliokoski, O. *et al.* Mice Do Not Habituate to Metabolism Cage Housing-A Three Week Study of Male BALB/c Mice. *PLoS One* **8**, (2013).

88. Kempermann, G., Brandon, E. P. & Gage, F. H. Environmental stimulation of 129/SvJ mice causes increased cell proliferation and neurogenesis in the adult dentate gyrus. *Curr. Biol.* **8**, 939–942 (1998).

89. Kemppinen, N. *et al.* Impact of aspen furniture and restricted feeding on activity, blood pressure, heart rate and faecal corticosterone and immunoglobulin A excretion in rats (Rattus norvegicus) housed in individually ventilated cages. *Lab. Anim.* **44**, 104–112 (2010).

90. Kentner, A. C., Lima, E., Migliore, M. M., Shin, J. & Scalia, S. Complex Environmental Rearing Enhances Social Salience and Affects Hippocampal Corticotropin Releasing Hormone Receptor Expression in a Sex-Specific Manner. *Neuroscience* **369**, 399–411 (2018).

91. Khalaji, S., Bigdeli, I., Ghorbani, R. & Miladi-Gorji, H. Environmental Enrichment Attenuates Morphine-Induced Conditioned Place Preference and Locomotor Sensitization in Maternally Separated Rat Pups. *Basic Clin. Neurosci.* **9**, 181–190 (2018).

92. Kim, M. S. *et al.* Environmental enrichment enhances synaptic plasticity by internalization of striatal dopamine transporters. *J. Cereb. Blood Flow Metab.* **36**, 2122–2133 (2016).

93. Kimura, L. F., Mattaraia, V. G. D. & Picolo, G. Distinct environmental enrichment protocols reduce anxiety but differentially modulate pain sensitivity in rats. *Behav. Brain Res.* **364**, 442–446 (2019).

94. Kirkpatrick, K., Marshall, A. T., Clarke, J. & Cain, M. E. Environmental Rearing Effects on Impulsivity and Reward Sensitivity. *Behav. Neurosci.* **127**, 712–724 (2013).

95. Klein, S. L., Lambert, K. G., Durr, D., Schaefer, T. & Waring, R. E. Influence of environmental enrichment and sex on predator stress response in rats. *Physiol Behav* **56**, 291–297 (1994).

96. Konkle, A. T. M., Kentner, A. C., Baker, S. L., Stewart, A. & Bielajew, C. Environmental-Enrichment-Related Variations in Behavioral, Biochemical, and Physiologic Responses of Sprague-Dawley and Long Evans Rats. *J. Am. Assoc. Lab. Anim. Sci.* **49**, 427–436 (2010).

97. Körholz, J. C. *et al.* Selective increases in inter-individual variability in response to environmental enrichment in female mice. *Elife* **7**, (2018).

98. Lach, G. *et al.* Short-term enriched environment exposure facilitates fear extinction in adult rats: The NPY-Y1 receptor modulation. *Neuropeptides* **55**, 73–78 (2016).

99. Lambert, K. *et al.* Natural-enriched environments lead to enhanced environmental engagement and altered neurobiological resilience. *Neuroscience* **330**, 386–394 (2016).

100. Larsson, F., Winblad, B. & Mohammed, A. H. Psychological stress and environmental adaptation in enriched vs. Impoverished housed rats. *Pharmacol. Biochem. Behav.* **73**, 193–207 (2002).

101. Latham, N. & Mason, G. Frustration and perseveration in stereotypic captive animals: Is a taste of enrichment worse than none at all? *Behav. Brain Res.* **211**, 96–104 (2010).

102. Lee, M. Y. *et al.* Alteration of synaptic activity-regulating genes underlying functional improvement by long-term exposure to an enriched environment in the adult brain. *Neurorehabil. Neural Repair* **27**, 561–574 (2013).

103. Leger, M. *et al.* Environmental enrichment improves recent but not remote memory in association with a modified brain metabolic activation profile in adult mice. *Behav. Brain Res.* **228**, 22–29 (2012).

104. Leger, M. *et al.* Environmental Enrichment Enhances Episodic-Like Memory in Association with a Modified Neuronal Activation Profile in Adult Mice. *PLoS One* **7**, (2012).

105. Lima, F. B. & Spinelli de Oliveira, E. What is the impact of low testosterone levels on the anatomical and behavioral repertoire of long-term enriched housing of male mice? *Behav. Processes* **108**, 57–64 (2014).

106. Lopes, D. A. *et al.* Anxiolytic and panicolytic-like effects of environmental enrichment seem to be modulated by serotonin neurons located in the dorsal subnucleus of the dorsal raphe. *Brain Res. Bull.* **150**, 272–280 (2019).

107. Lores-Arnaiz, S. *et al.* Extensive enriched environments protect old rats from the aging dependent impairment of spatial cognition, synaptic plasticity and nitric oxide production. *Behav. Brain Res.* **169**, 294–302 (2006).

108. Lores-Arnaiz, S. *et al.* Exposure to enriched environments increases brain nitric oxide synthase and improves cognitive performance in prepubertal but not in young rats. *Behav. Brain Res.* **184**, 117–123 (2007).

109. Loss, C. M. *et al.* Influence of environmental enrichment vs. time-of-day on behavioral repertoire of male albino Swiss mice. *Neurobiol. Learn. Mem.* **125**, 63–72 (2015).

110. Lukkes, J. L., Mokin, M. V, Scholl, J. L. & Forster, G. L. Adult rats exposed to early-life social isolation exhibit increased anxiety and conditioned fear behavior, and altered hormonal stress responses. *Horm. Behav.* **55**, 248–256 (2009).

111. Makowska, I. J. & Weary, D. M. The importance of burrowing, climbing and standing upright for laboratory rats. *R. Soc. Open Sci.* **3**, (2016).

112. Makowska, I. J. & Weary, D. M. Differences in Anticipatory Behaviour between Rats (Rattus norvegicus) Housed in Standard versus Semi-Naturalistic Laboratory Environments. *PLoS One* **11**, (2016).

113. Manno, F. A. M. *et al.* Environmental enrichment leads to behavioral circadian shifts enhancing brain-wide functional connectivity between sensory cortices and eliciting increased hippocampal spiking. *Neuroimage* **252**, (2022).

114. Manosevitz, M. & Joel, U. Behavioral effects of environmental enrichment in randomly bred mice. *J. Comp. Physiol. Psychol.* **85**, 373–382 (1973).

115. Marashi, V., Barnekow, A., Ossendorf, E. & Sachser, N. Effects of different forms of environmental enrichment on behavioral, endocrinological, and immunological parameters in male mice. *Horm. Behav.* **43**, 281–292 (2003).

116. Marashi, V., Barnekow, A. & Sachser, N. Effects of environmental enrichment on males of a docile inbred strain of mice. *Physiol. Behav.* **82**, 765–776 (2004).

117. Mármol, F., Sánchez, J., Torres, M. N. & Chamizo, V. D. Environmental enrichment in the absence of wheel running produces beneficial behavioural and anti-oxidative effects in rats. *Behav Process.* **144**, 66–71 (2017).

118. Marques, J. M. & Olsson, I. A. S. The effect of preweaning and postweaning housing on the behaviour of the laboratory mouse (Mus musculus). *Lab. Anim.* **41**, 92–102 (2007).

119. Martinez, A. R., Brunelli, S. A. & Zimmerberg, B. Communal nesting exerts epigenetic influences on affective and social behaviors in rats selectively bred for an infantile trait. *Physiol Behav* **139**, 97–103 (2015).

120. McQuaid, R. J., Audet, M. C. & Anisman, H. Environmental enrichment in male CD-1 mice promotes aggressive behaviors and elevated corticosterone and brain norepinephrine activity in response to a mild stressor. *Stress. Int. J. Biol. Stress* **15**, 354–360 (2012).

121. McQuaid, R. J., Audet, M. C., Jacobson-Pick, S. & Anisman, H. The differential impact of social defeat on mice living in isolation or groups in an enriched environment: plasma corticosterone and monoamine variations. *Int. J. Neuropsychopharmacol.* **16**, 351–363 (2013).

122. Melani, R., Chelini, G., Cenni, M. C. & Berardi, N. Enriched environment effects on remote object recognition memory. *Neuroscience* **352**, 296–305 (2017).

123. Melendez, R. I., Gregory, M. L., Bardo, M. T. & Kalivas, P. W. Impoverished rearing environment alters metabotrophic glutamate receptor expression and function in the prefrontal cortex. *Neuropsychopharmacology* **29**, 1980–1987 (2004).

124. Mendes, F., da Paixao, L., Diniz, C. W. P. & Sosthenes, M. C. K. Environmental Impoverishment, Aging, and Reduction in Mastication Affect Mouse Innate Repertoire to Explore Novel Environments and to Assess Risk. *Front. Neurosci.* **13**, (2019).

125. Mesa-Gresa, P., Perez-Martinez, A. & Redolat, R. Environmental Enrichment Improves Novel Object Recognition and Enhances Agonistic Behavior in Male Mice. *Aggress. Behav.* **39**, 269–279 (2013).

126. Mesa-Gresa, P., Ramos-Campos, M. & Redolat, R. Corticosterone levels and behavioral changes induced by simultaneous exposure to chronic social stress and enriched environments in NMRI male mice. *Physiol. Behav.* **158**, 6–17 (2016).

127. Mitchell, E. N., Marston, H. M., Nutt, D. J. & Robinson, E. S. J. Evaluation of an operant successive negative contrast task as a method to study affective state in rodents. *Behav. Brain Res.* **234**, 155–160 (2012).

128. Modlinska, K., Chrzanowska, A. & Pisula, W. Variability of enriched environment does not enhance the enrichment effect on food neophobia in rats (Rattus norvegicus). *Behav. Processes* **180**, (2020).

129. Modlinska, K., Chrzanowska, A. & Pisula, W. The impact of changeability of enriched environment on exploration in rats. *Behav. Processes* **164**, 78–85 (2019).

130. Mogensen, J. Influences of the rearing conditions on functional properties of the rat’s prefrontal system. *Behav. Brain Res.* **42**, 135–142 (1991).

131. Mohammed, A. K., Winblad, B., Ebendal, T. & Larkfors, L. Environmental influence on behaviour and nerve growth factor in the brain. *Brain Res.* **528**, 62–72 (1990).

132. Molina, S. J., Lietti, Á. E., Carreira Caro, C. S., Buján, G. E. & Guelman, L. R. Effects of early noise exposure on hippocampal-dependent behaviors during adolescence in male rats: influence of different housing conditions. *Anim. Cogn.* **25**, 103–120 (2022).

133. Mora-Gallegos, A. *et al.* Age-dependent effects of environmental enrichment on spatial memory and neurochemistry. *Neurobiol. Learn. Mem.* **118**, 96–104 (2015).

134. Mosaferi, B., Babri, S., Ebrahimi, H. & Mohaddes, G. Enduring effects of post-weaning rearing condition on depressive- and anxiety-like behaviors and motor activity in male rats. *Physiol. Behav.* **142**, 131–136 (2015).

135. Mustroph, M. L. *et al.* Aerobic exercise is the critical variable in an enriched environment that increases hippocampal neurogenesis and water maze learning in male C57BL/6J mice. *Neuroscience* **219**, 62–71 (2012).

136. Nikolaev, E., Kaczmarek, L., Zhu, S. W., Winblad, B. & Mohammed, A. H. Environmental manipulation differentially alters c-Fos expression in amygdaloid nuclei following aversive conditioning. *Brain Res.* **957**, 91–98 (2002).

137. Nilsson, L. *et al.* Environmental influence on somatostatin levels and gene expression in the rat brain. *Brain Res.* **628**, 93–98 (1993).

138. Nip, E. *et al.* Why are enriched mice nice? Investigating how environmental enrichment reduces agonism in female C57BL/6, DBA/2, and BALB/c mice. *Appl. Anim. Behav. Sci.* **217**, 73–82 (2019).

139. Nishijima, T. *et al.* Cessation of voluntary wheel running increases anxiety-like behavior and impairs adult hippocampal neurogenesis in mice. *Behav. Brain Res.* **245**, 34–41 (2013).

140. Núñez-Murrieta, M. A. *et al.* Maternal behavior, novelty confrontation, and subcortical c-Fos expression during lactation period are shaped by gestational environment. *Behav. Brain Res.* **412**, (2021).

141. Oatess, T. L., Harrison, F. E., Himmel, L. E. & Jones, C. P. Effects of acrylic tunnel enrichment on anxiety-like behavior, neurogenesis, and physiology of C57BL/6J Mice. *J. Am. Assoc. Lab. Anim. Sci.* **60**, 44–53 (2021).

142. Paré, W. P. & Kluczynski, J. Developmental factors modify stress ulcer incidence in a stress-susceptible rat strain. in *Journal of Physiology Paris* **91**, 105–111 (1997).

143. Park, G. A. S., Pappas, B. A., Murtha, S. M. & Ally, A. Enriched environment primes forebrain choline acetyltransferase activity to respond to learning experience. *Neurosci. Lett.* **143**, 259–262 (1992).

144. Pawlowicz, A., Demner, A. & Lewis, M. H. Effects of access to voluntary wheel running on the development of stereotypy. *Behav Process.* **83**, 242–246 (2010).

145. Paylor, R., Morrison, S. K., Rudy, J. W., Waltrip, L. T. & Wehner, J. M. Brief exposure to an enriched environment improves performance on the Morris water task and increases hippocampal cytosolic protein kinase C activity in young rats. *Behav. Brain Res.* **52**, 49–56 (1992).

146. Pena, Y., Prunell, M., Rotllant, D., Armario, A. & Escorihuela, R. M. Enduring effects of environmental enrichment from weaning to adulthood on pituitary-adrenal function, pre-pulse inhibition and learning in male and female rats. *Psychoneuroendocrinology* **34**, 1390–1404 (2009).

147. Pham, T. M., Brené, S. & Baumans, V. Behavioral assessment of intermittent wheel running and individual housing in mice in the laboratory. *J Appl Anim Welf Sci* **8**, 157–173 (2005).

148. Pham, T. M. *et al.* Changes in brain nerve growth factor levels and nerve growth factor receptors in rats exposed to environmental enrichment for one year. *Neuroscience* **94**, 279–286 (1999).

149. Pietropaolo, S. *et al.* Long-term effects of the periadolescent environment on exploratory activity and aggressive behaviour in mice: social versus physical enrichment. *Physiol. Behav.* **81**, 443–453 (2004).

150. Pietropaolo, S., Feldon, J., Alleva, E., Cirulli, F. & Yee, B. K. The role of voluntary exercise in enriched rearing: A behavioral analysis. *Behav. Neurosci.* **120**, 787–803 (2006).

151. Pinelli, C. J., Leri, F. & Turner, P. V. Long Term Physiologic and Behavioural Effects of Housing Density and Environmental Resource Provision for Adult Male and Female Sprague Dawley Rats. *Animals* **7**, (2017).

152. Plenz, U. T. & Kanold, P. O. Differences in running performance of single- and group-housed mice. (2021). doi:10.1101/2021.12.29.474296

153. Pöǧün, S., Kanit, L. & Okur, B. E. Learning-induced changes in D2 receptors of rat brain are sexually dimorphic. *Pharmacol. Biochem. Behav.* **43**, 71–75 (1992).

154. Prusky, G. T., Reidel, C. & Douglas, R. M. Environmental enrichment from birth enhances visual acuity but not place learning in mice. *Behav. Brain Res.* **114**, 11–15 (2000).

155. Rabadan, R., Ramos-Campos, M., Redolat, R. & Mesa-Gresa, P. Physical activity and environmental enrichment: Behavioural effects of exposure to different housing conditions in mice. *Acta Neurobiol. Exp. (Wars).* **79**, 374–385 (2019).

156. Rochefort, C., Gheusi, G., Vincent, J. D. & Lledo, P. M. Enriched odor exposure increases the number of newborn neurons in the adult olfactory bulb and improves odor memory. *J Neurosci* **22**, 2679–2689 (2002).

157. Rojas-Carvajal, M., Fornaguera, J., Mora-Gallegos, A. & Brenes, J. C. Testing experience and environmental enrichment potentiated open-field habituation and grooming behaviour in rats. *Anim. Behav.* **137**, 225–235 (2018).

158. Rojas-Carvajal, M., Sequeira-Cordero, A. & Brenes, J. C. Neurobehavioral Effects of Restricted and Unpredictable Environmental Enrichment in Rats. *Front. Pharmacol.* **11**, (2020).

159. Rose, F. D., Love, S. & Dell, P. A. Differential reinforcement effects in rats reared in enriched and impoverished environments. *Physiol. Behav.* **36**, 1139–1145 (1986).

160. Rossi, H. L. & Neubert, J. K. Effects of environmental enrichment on thermal sensitivity in an operant orofacial pain assay. *Behav. Brain Res.* **187**, 478–482 (2008).

161. Ros-Simó, C. & Valverde, O. Early-life social experiences in mice affect emotional behaviour and hypothalamic-pituitary-adrenal axis function. *Pharmacol. Biochem. Behav.* **102**, 434–441 (2012).

162. Rountree-Harrison, D., Burton, T. J., Leamey, C. A. & Sawatari, A. Environmental Enrichment Expedites Acquisition and Improves Flexibility on a Temporal Sequencing Task in Mice. *Front. Behav. Neurosci.* **12**, (2018).

163. Roy, V., Belzung, C., Delarue, C. & Chapillon, P. Environmental enrichment in BALB/c mice - Effects in classical tests of anxiety and exposure to a predatory odor. *Physiol. Behav.* **74**, 313–320 (2001).

164. Salinas-Velarde, I. D. *et al.* Lower ΔFosB expression in the dopaminergic system after stevia consumption in rats housed under environmental enrichment conditions. *Brain Res. Bull.* **177**, 172–180 (2021).

165. Sampedro-Piquero, P., Arias, J. L. & Begega, A. Behavioral testing-related changes in the expression of Synapsin I and glucocorticoid receptors in standard and enriched aged Wistar rats. *Exp Gerontol* **58**, 292–302 (2014).

166. Sampedro-Piquero, P., Begega, A., Zancada-Menendez, C., Cuesta, M. & Arias, J. L. Age-dependent effects of environmental enrichment on brain networks and spatial memory in Wistar rats. *Neuroscience* **248**, 43–53 (2013).

167. Sampedro-Piquero, P., Zancada-Menendez, C. & Begega, A. Housing condition-related changes involved in reversal learning and its c-Fos associated activity in the prefrontal cortex. *Neuroscience* **307**, 14–25 (2015).

168. Schrijver, N. C. A., Pallier, P. N., Brown, V. J. & Wurbel, H. Double dissociation of social and environmental stimulation on spatial learning and reversal learning in rats. *Behav. Brain Res.* **152**, 307–314 (2004).

169. Schrijver, N. C. A., Bahr, N. I., Weiss, I. C. & Würbel, H. Dissociable effects of isolation rearing and environmental enrichment on exploration, spatial learning and HPA activity in adult rats. *Pharmacol. Biochem. Behav.* **73**, 209–224 (2002).

170. Sequeira-Cordero, A., Mora-Gallegos, A., Cuenca-Berger, P. & Fornaguera-Trías, J. Individual differences in the forced swimming test and the effect of environmental enrichment: Searching for an interaction. *Neuroscience* **265**, 95–107 (2014).

171. Serradj, N. & Jamon, M. Postnatal training of 129/Sv mice confirms the long-term influence of early exercising on the motor properties of mice. *Behav. Brain Res.* **310**, 126–134 (2016).

172. Seward, T., Harfmann, B. D., Esser, K. A. & Schroder, E. A. Reinventing the wheel: comparison of two wheel cage styles for assessing mouse voluntary running activity. *J Appl Physiol* **124**, 923–929 (2018).

173. Sherman, G. F. & Galaburda, A. M. Neocortical asymmetry and open-field behavior in the rat. *Exp. Neurol.* **86**, 473–482 (1984).

174. Silva, C. F., Duarte, F. S., Lima, T. C. M. De & De Oliveira, C. L. Effects of social isolation and enriched environment on behavior of adult Swiss mice do not require hippocampal neurogenesis. *Behav. Brain Res.* **225**, 85–90 (2011).

175. Simonetti, T., Lee, H., Bourke, M., Leamey, C. A. & Sawatari, A. Enrichment from birth accelerates the functional and cellular development of a motor control area in the mouse. *PLoS One* **4**, (2009).

176. Soeda, F. *et al.* Effects of enriched environment on micturition activity in freely moving C57BL/6J mice. *LUTS Low. Urin. Tract Symptoms* **13**, 400–409 (2021).

177. Song, S. Y. *et al.* Environmental Enrichment Upregulates Striatal Synaptic Vesicle-Associated Proteins and Improves Motor Function. *Front. Neurol.* **9**, (2018).

178. Sorensen, D. B., Mortensen, K., Bertelsen, T. & Vognbjer, K. Enriching the metabolic cage: effects on rat physiology and behaviour. *Anim. Welf.* **17**, 395–403 (2008).

179. Spangenberg, E. M. F., Augustsson, H., Dahlborn, K., Essen-Gustavsson, B. & Cvek, K. Housing-related activity in rats: effects on body weight, urinary corticosterone levels, muscle properties and performance. *Lab. Anim.* **39**, 45–57 (2005).

180. Sparling, J. E., Mahoney, M., Baker, S. & Bielajew, C. The effects of gestational and postpartum environmental enrichment on the mother rat: A preliminary investigation. *Behav. Brain Res.* **208**, 213–223 (2010).

181. Sparling, J. E., Baker, S. L. & Bielajew, C. Effects of combined pre- and post-natal enrichment on anxiety-like, social, and cognitive behaviours in juvenile and adult rat offspring. *Behav. Brain Res.* **353**, 40–50 (2018).

182. Spencer, P. J., Mattsson, J. L., Johnson, K. A. & Albee, R. R. Neurotoxicity screening methods are sensitive to experimental history. *Int. J. Psychophysiol.* **14**, 5–19 (1993).

183. Swanson, H. H., McConnell, P., Uylings, H. B., Van Oyen, H. G. & Van de Poll, N. E. Interaction between pre-weaning undernutrition and post-weaning environmental enrichment on somatic development and behaviour in male and female rats. *Behav Process.* **8**, 1–20 (1983).

184. Teather, L. A., Magnusson, J. E., Chow, C. M. & Wurtman, R. J. Environmental conditions influence hippocampus-dependent behaviours and brain levels of amyloid precursor protein in rats. *Eur. J. Neurosci.* **16**, 2405–2415 (2002).

185. Tees, R. C. The influences of rearing environment and neonatal choline dietary supplementation on spatial learning and memory in adult rats. *Behav. Brain Res.* **105**, 173–188 (1999).

186. Tees, R. C. The influences of sex, rearing environment, and neonatal choline dietary supplementation on spatial and nonspatial learning and memory in adult rats. *Dev. Psychobiol.* **35**, 328–342 (1999).

187. Tilly, S. L. C., Dallaire, J. & Mason, G. J. Middle-aged mice with enrichment-resistant stereotypic behaviour show reduced motivation for enrichment. *Anim. Behav.* **80**, 363–373 (2010).

188. Ulupinar, E., Erol, K., Aya, H. & Yucel, F. Rearing conditions differently affect the motor performance and cerebellar morphology of prenatally stressed juvenile rats. *Behav. Brain Res.* **278**, 235–243 (2015).

189. Urakawa, S., Mitsushima, D., Shimozuru, M., Sakuma, Y. & Kondo, Y. An Enriched Rearing Environment Calms Adult Male Rat Sexual Activity: Implication for Distinct Serotonergic and Hormonal Responses to Females. *PLoS One* **9**, (2014).

190. Urakawa, S. *et al.* Rearing in enriched environment increases parvalbumin-positive small neurons in the amygdala and decreases anxiety-like behavior of male rats. *Bmc Neurosci.* **14**, (2013).

191. Vakhnin, V. A. & Bryukhin, G. V. Effects of Environmental Conditions on Behavior in an Open Field Test in Rats Born to Females with Chronic Alcoholization. *Neurosci. Behav. Physiol.* **45**, 1003–1009 (2015).

192. Van de Weerd, H. A. *et al.* Effects of environmental enrichment for mice: Variation in experimental results. *J. Appl. Anim. Welf. Sci.* **5**, 87–109 (2002).

193. Van De Weerd, H. A., Van Loo, P. L. P., Van Zutphen, L. F. M., Koolhaas, J. M. & Baumans, V. Nesting material as environmental enrichment has no adverse effects on behavior and physiology of laboratory mice. *Physiol. Behav.* **62**, 1019–1028 (1997).

194. van der Geest, J. N., Spoor, M. & Frens, M. A. Environmental Enrichment Improves Vestibular Oculomotor Learning in Mice. *Front. Behav. Neurosci.* **15**, (2021).

195. VanWaas, M. & Soffie, M. Differential environmental modulations on locomotor activity, exploration and spatial behaviour in young and old rats. *Physiol. Behav.* **59**, 265–271 (1996).

196. Varman, D. R. & Rajan, K. E. Environmental Enrichment Reduces Anxiety by Differentially Activating Serotonergic and Neuropeptide Y (NPY)-Ergic System in Indian Field Mouse (Mus booduga): An Animal Model of Post-Traumatic Stress Disorder. *PLoS One* **10**, (2015).

197. Vazquez-Sanroman, D. B., Wilson, G. A. & Bardo, M. T. Effects of Social Isolation on Perineuronal Nets in the Amygdala Following a Reward Omission Task in Female Rats. *Mol. Neurobiol.* **58**, 348–361 (2021).

198. Vedovelli, K. *et al.* Effects of increased opportunity for physical exercise and learning experiences on recognition memory and brain-derived neurotrophic factor levels in brain and serum of rats. *Neuroscience* **199**, 284–291 (2011).

199. Vivinetto, A. L., Suarez, M. M. & Rivarola, M. A. Neurobiological effects of neonatal maternal separation and post-weaning environmental enrichment. *Behav. Brain Res.* **240**, 110–118 (2013).

200. Wainwright, P. E., Huang, Y. S., Bulman-Fleming, B., Lévesque, S. & McCutcheon, D. The effects of dietary fatty acid composition combined with environmental enrichment on brain and behavior in mice. *Behav. Brain Res.* **60**, 125–136 (1994).

201. Walasek, G., Wesierska, M. & Werka, T. Effects of social rearing conditions on conditioned suppression in rats. *Acta Neurobiol. Exp. (Wars).* **62**, 25–31 (2002).

202. Wang, L. *et al.* Enriched Physical Environment Attenuates Spatial and Social Memory Impairments of Aged Socially Isolated Mice. *Int. J. Neuropsychopharmacol.* **21**, 1114–1127 (2018).

203. Wang, R. X., Hausknecht, K. A., Haj-Dahmane, S., Shen, R. Y. & Richards, J. B. Decreased environmental complexity during development impairs habituation of reinforcer effectiveness of sensory stimuli. *Behav. Brain Res.* **337**, 53–60 (2018).

204. Wheeler, R. R., Swan, M. P. & Hickman, D. L. Effect of multilevel laboratory rat caging system on the well-being of the singly-housed Sprague Dawley rat. *Lab. Anim.* **49**, 10–19 (2015).

205. Winocur, G. Environmental influences on cognitive decline in aged rats. *Neurobiol. Aging* **19**, 589–597 (1998).

206. Winocur, G. & Greenwood, C. E. The effects of high fat diets and environmental influences on cognitive performance in rats. *Behav. Brain Res.* **101**, 153–161 (1999).

207. Wirz, A., Mandillo, S., D’Amato, F. R., Giuliani, A. & Riviello, M. C. Response, use and habituation to a mouse house in C57BL/6J and BALB/c mice. *Exp. Anim.* **64**, 281–293 (2015).

208. Wolfer, D. P. *et al.* Laboratory animal welfare: cage enrichment and mouse behaviour. *Nature* **432**, 821–822 (2004).

209. Wood, D. A. & Rebec, G. V. Environmental enrichment alters neuronal processing in the nucleus accumbens core during appetitive conditioning. *Brain Res.* **1259**, 59–67 (2009).

210. Wood, D. A., Siegel, A. K. & Rebec, G. V. Environmental enrichment reduces impulsivity during appetitive conditioning. *Physiol. Behav.* **88**, 132–137 (2006).

211. Workman, J. L., Fonken, L. K., Gusfa, J., Kassouf, K. M. & Nelson, R. J. Post-weaning environmental enrichment alters affective responses and interacts with behavioral testing to alter nNOS immunoreactivity. *Pharmacol Biochem Behav* **100**, 25–32 (2011).

212. Würbel, H., Chapman, R. & Rutland, C. Effect of feed and environmental enrichment on development of stereotypic wire-gnawing in laboratory mice. *Appl. Anim. Behav. Sci.* **60**, 69–81 (1998).

213. Xie, H. Y. *et al.* Enrichment-induced exercise to quantify the effect of different housing conditions: A tool to standardize enriched environment protocols. *Behav. Brain Res.* **249**, 81–89 (2013).

214. Zaias, J., Queeney, T. J., Kelley, J. B., Zakharova, E. S. & Izenwasser, S. Social and physical environmental enrichment differentially affect growth and activity of preadolescent and adolescent male rats. *J. Am. Assoc. Lab. Anim. Sci.* **47**, 30–34 (2008).

215. Zakharova, E., Starosciak, A., Wade, D. & Izenwasser, S. Sex differences in the effects of social and physical environment on novelty-induced exploratory behavior and cocaine-stimulated locomotor activity in adolescent rats. *Behav. Brain Res.* **230**, 92–99 (2012).

216. Zanca, R. M. *et al.* Environmental enrichment increases glucocorticoid receptors and decreases GluA2 and protein kinase M Zeta (PKMζ) trafficking during chronic stress: A protective mechanism? *Front. Behav. Neurosci.* **9**, (2015).

217. Zeleznikow-Johnston, A., Burrows, E. L., Renoir, T. & Hannan, A. J. Environmental enrichment enhances cognitive flexibility in C57BL/6 mice on a touchscreen reversal learning task. *Neuropharmacology* **117**, 219–226 (2017).

218. Zhu, S. W. *et al.* Neurotrophin levels and behaviour in BALB/c mice: Impact of intermittent exposure to individual housing and wheel running. *Behav. Brain Res.* **167**, 1–8 (2006).
